# Alpine bogs of southern Spain show human-induced environmental change superimposed on long-term natural variations

**DOI:** 10.1038/s41598-017-07854-w

**Published:** 2017-08-07

**Authors:** Antonio García-Alix, Francisco J. Jiménez-Espejo, Jaime L. Toney, Gonzalo Jiménez-Moreno, María J. Ramos-Román, R. Scott Anderson, Patricia Ruano, Ignasi Queralt, Antonio Delgado Huertas, Junichiro Kuroda

**Affiliations:** 1 School of Geographical and Earth Sciences, University of Glasgow, Glasgow, UK; 2 Departamento de Estratigrafía y Paleontología, Universidad de Granada, Granada, Spain; 3 Department of Biogeochemistry (JAMSTEC), Yokosuka, Japan; 4 School of Earth Sciences and Environmental Sustainability, Northern Arizona University, Flagstaff, AZ USA; 5 Departamento de Geodinámica, Universidad de Granada, Granada, Spain; 6 Instituto Andaluz de Ciencias de la Tierra (IACT), CISC-UGR Armilla, Spain; 7 Institute of Environmental Assessment and Water Research (IDAEA), CSIC Barcelona, Spain

**Keywords:** Biogeochemistry, Biogeochemistry, Limnology

## Abstract

Recent studies have proved that high elevation environments, especially remote wetlands, are exceptional ecological sensors of global change. For example, European glaciers have retreated during the 20^th^ century while the Sierra Nevada National Park in southern Spain witnessed the first complete disappearance of modern glaciers in Europe. Given that the effects of climatic fluctuations on local ecosystems are complex in these sensitive alpine areas, it is crucial to identify their long-term natural trends, ecological thresholds, and responses to human impact. In this study, the geochemical records from two adjacent alpine bogs in the protected Sierra Nevada National Park reveal different sensitivities and long-term environmental responses, despite similar natural forcings, such as solar radiation and the North Atlantic Oscillation, during the late Holocene. After the Industrial Revolution both bogs registered an independent, abrupt and enhanced response to the anthropogenic forcing, at the same time that the last glaciers disappeared. The different response recorded at each site suggests that the National Park and land managers of similar regions need to consider landscape and environmental evolution in addition to changing climate to fully understand implications of climate and human influence.

## Introduction

Mountainous areas in the Mediterranean region are among the most vulnerable in Europe, and have been severely affected by recent climate and human-induced environmental changes with regard to water resources, temperature gradients, sensitive species, and soil fertility^1, 2^. Alpine systems here function as ‘sky islands’, because plant communities are often patchy, occur over narrow elevational bands and are highly susceptible to environmental and climate stressors. The lack of ecosystem connectivity leaves plant communities and plant species (many of which are endemic) highly vulnerable to extinction^2–4^, because they cannot respond via latitudinal redistribution, but shift altitudinally^5–7^.

The long-term natural environmental evolution in the western Mediterranean region follows an aridification and desertification trend during the late Holocene^8–11^. This signal has been influenced and even boosted by human impact, especially in the last centuries^11–13^. So far, the increased rate and magnitude of recent changes have had significant economic and social consequences, such as crop destruction, human migrations, or conflicts between farmers and herders^14^. However, exactly how these recent fluctuations will manifest in additional environmental changes is still largely unknown, so longer-term records that demonstrate how current changes fit into the historical context are needed. These longer-term records will help in understanding future change, where climatic forecasts for the mountains of the Mediterranean region are not optimistic, and point toward a marked temperature increase and precipitation decrease^15^, which are likely to accelerate environmental degradation.

**Figure 1:**
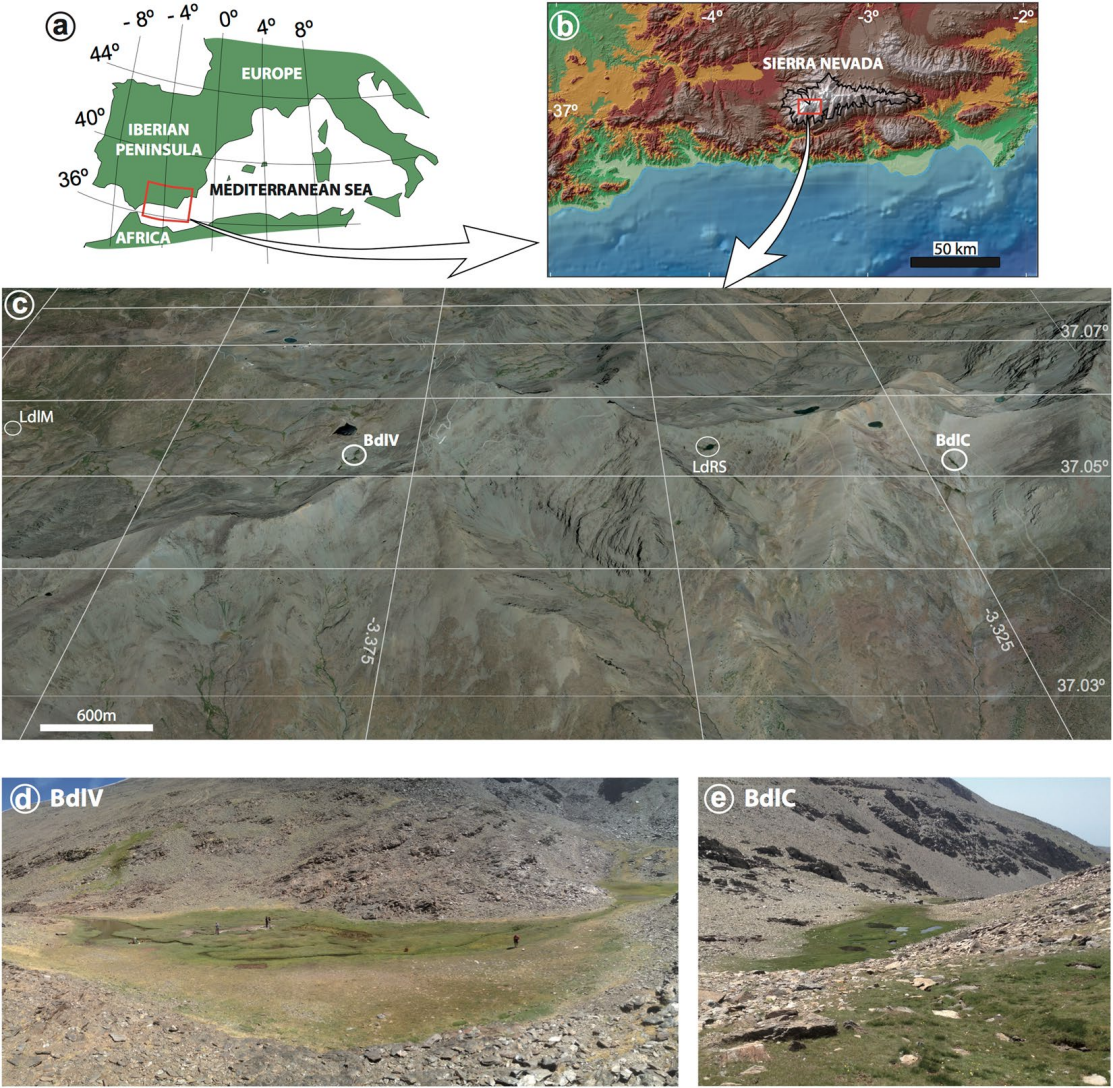
Geographical setting. (**a**) Location of the studied area in the western Mediterranean, (**b**) the relief of Sierra Nevada (black line, National Park limit), and (**c**) the location of studied sedimentary records, including the sampling areas for vegetation and soils, Laguna de la Mula (LdlM), Borreguil de la Virgen (BdlV), Laguna de Río Seco (LdRS), and Borreguil de la Caldera site (BdlC), (**d**) Borreguil de la Virgen area and (**e**) Borreguil de la Caldera area. Data source and software: (**a**) map created by P. Ruano using Adobe Illustrator [5.5] (https://www.adobe.com/), (**b**) data from Suttle Radar Tomography Mission (SRTM-90: http://www2.jpl.nasa.gov/srtm/)^65^ plotted by means of ArcMap [10.1] (http://www.esri.com/software/arcgis/arcgis-for-desktop), (**c**) map from Google Earth Pro [7.1.5.1557] (https://www.google.es/earth/download/gep/agree.html) using the data provided by Google 2016 and DigitalGlobe 2016, and (**d,e**) pictures from G. Jiménez-Moreno.

The highest elevation environments in the Sierra Nevada of southern Iberia (∼3000 masl) have experienced little direct impact from human activities for centuries. Although these areas are actively protected today due to their environmental richness, and are part of the Natural Park and Biosphere Reserve of Sierra Nevada^16^, they have recorded indirect influence of human activities such as reforestation and atmospheric pollution^9, 13^. Wetlands in this alpine area are mainly oligotrophic, since their catchment basins consist of bare mica-schist bedrock with sparse soil development. Consequently, their biogeochemistry is highly influenced by allocthonous atmospheric inputs that supply important nutrients rather than by local sources^17, 18^. All of these features make these remote alpine environments outstanding ecological observatories of climate change^19^ that allow the identification of the signatures of both natural and human-induced environmental changes^9, 10, 13, 18^. In particular, our previous research from sedimentary archives of these wetlands shows a possible increase in the rate of environmental change during the last century^10, 20^, in agreement with the sharp precipitation decrease and temperature increase, as well as an increase in industrial activities and changes in land use in the western Mediterranean region^15, 21–24^. Clear evidence of these changes include the final melting of the Little Ice Age (LIA) Corral del Veleta Glacier (the southernmost glacier in Europe) in the 1920s^25^ and the gradual permafrost reduction in recent decades^26^. In this paper we identify the oscillations and trends of natural and anthropogenically-induced ecosystem changes by comparing two adjacent Holocene records from high elevation wetlands in the Sierra Nevada: Borreguil de la Virgen (BdlV: 37° 03′ 15″ N; 3° 22′ 40″ W) and Borreguil de la Caldera (BdlC: 37° 03′ 02″ N; 3° 19′ 24″ W) (Fig. 1). The sediments mainly consist of clays and peat in both sites, but the sedimentary record is longer in BdlV (165 cm, ~8.5 cal ky BP)^27^ than in BdlC (56 cm, ~4.5 cal ky BP)^28^. The sedimentary records along with the age models of both sites are depicted in Supplementary Fig. S1. The Holocene pollen record in both sites^27, 28^, along with the organic data from bulk sediment (carbon and nitrogen) in BdlV^20^ have provided a strong framework to design the present study. The outcomes of this work can be used to set up strategies to minimise the impact of present abrupt climate changes in sensitive areas and to identify other potentially vulnerable high-elevation sites.

The selected high-elevation peat bogs (called “borreguiles”) have similar areas (∼0.18 hectares in BdlV and ∼0.17 hectares in BdlC) and elevation (2945 masl in BdlV and 2992 masl in BdlC), but the catchment basins and valley orientations are different (Fig. 1). The catchment basin of BdlV is ∼30 hectares, and it is located in the north-west-facing Dílar River Valley. However, the catchment basin of BdlC, which is nearly twice the size, ∼62 hectares, is located in the south-facing glacially carved Poqueira River Valley. Most of the alpine catchment basins in the Sierra Nevada area are small and non-vegetated, with plant growth limited to the bog surface and immediate surrounding wetland during the ice-free season (from ∼April to ∼October)^29^. The small size of the catchment basins and peatland areas as well as the effect of the steep topography, imply that our records not only register the environmental evolution of the peatland areas, but could also be influenced by their catchment basins.

Vegetation in Sierra Nevada is distributed in elevationally-determined belts, controlled by the amount of precipitation and seasonal temperature gradients^9^. The present treeline occurs at ∼2550 masl and the selected records are located in the tundra-like vegetation belt with open herbaceous grasslands (above ∼2900 masl)^9, 29^. Peatland vegetation mainly consists of Poaceae and Cyperaceae, as well as non-vascular plants (bryophytes), even though there are also some less abundant plant taxa^29–31^. The studied sites are mostly ombrotrophic terrestrial peatlands, since the primary hydrological input to both bogs is from direct precipitation (rain or snow). However, BdlC has a small spring upstream that is fed through groundwater seepage from Laguna de la Caldera, located at the head of the catchment basin at ∼3030 masl (Fig. 1c).

## Results

### Recent plant and soil biomarkers

One way to detect changes in vegetation and vegetation belts through time is by analysis of *n*-alkanes from plant leaf waxes. Investigation of the distribution of different chain lengths in modern plant species^32–34^ can be applied to interpretations of past vegetation, environmental and landscape changes^35^. Our modern plant and soil survey in the extreme Sierra Nevada environments (Fig. 1c) shows that the distance plants occur from a water source, such as wetlands, controls the length of *n-*alkanes carbon chains. In particular, plants that are in or near the water pools show a stronger predominance of the shorter carbon chains. This relationship is expressed by different *n-*alkane indices, such as average chain length (ACL), carbon preference index (CPI), and proportion of aquatics (P_aq_) in the studied sites (Table 1; Supplementary Fig. S2). ACL values and the most abundant *n-*alkane are usually lower in areas closer to the water pools. Opposite trends have been found in the P_aq_. CPI values are higher than 3, which indicate that diagenesis and thermal alteration have not occurred^32^. Analysis of modern plant CPI values show that the lowest values are recorded in algae/moss/peat samples, as expected (Table 1; Supplementary Fig. S2).

**Table:** Summary of the *n-*alkane indices (CPI, ACL, and P_aq_) from the studied plant, algae and peat samples at different distance from the water pools in Sierra Nevada (see more details in Supplementary Fig. S2).

Distance from the main water pool	*n-*alkane indices		
	CPI	ACL	P_aq_
Far	22.0 ± 10.4	30.2 ± 1.0	0.02 ± 0.02
Intermediate	13.6 ± 8.6	29.8 ± 0.4	0.10 ± 0.03
Near/in	6.5 ± 2.5	28.7 ± 0.5	0.32 ± 0.12

### Organic geochemistry in the sedimentary records

**Figure 2:**
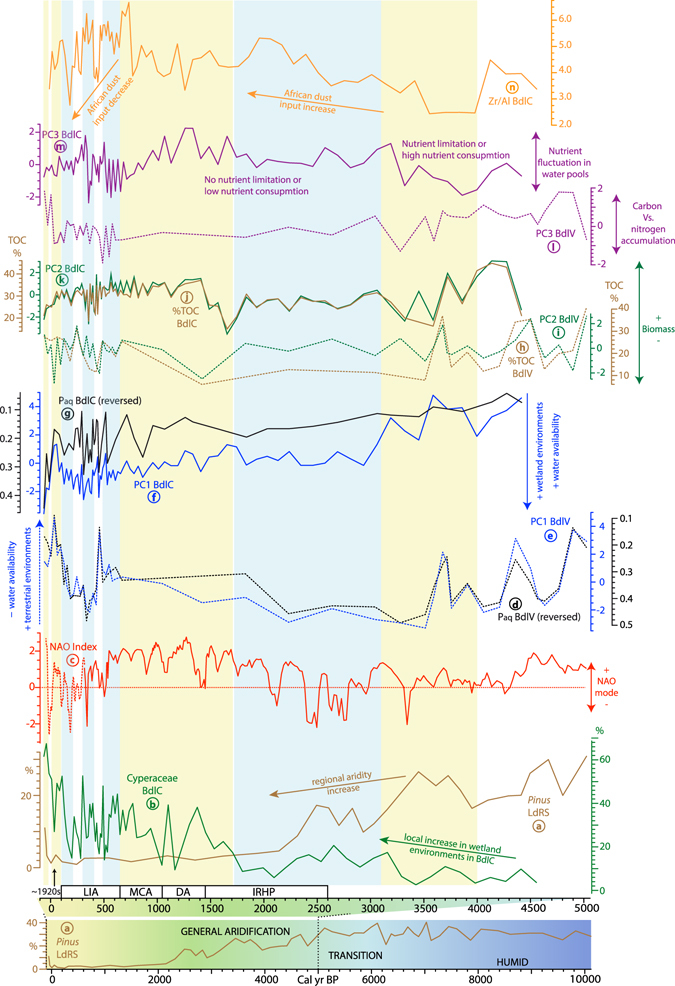
Comparison of humidity trends along with different productivity features and African dust input fluctuations during the middle-to-late Holocene in the studied records, including: (**a**) general trends of humidity in the studied region along with Pinus pollen record of LdRS^9, 10, 20, 27, 28, 57^ (blue colour: humid periods; yellow colours: dry periods), (**b**) local wetland development at BdlC deduced from Cyperaceae pollen^28^, (**c**) middle-to-late Holocene NAO index reconstruction: solid line^45^ and dotted line^49^, (**d–g**) water availability evolution in both cores (PC1s and P_aq_), (**h–k**) biomass accumulation/production in both cores (PC2s and %TOC), (**l**) terrestrial/algal inputs in the bog (PC3 of BdlV), (**m**) isotopic fluctuations caused by nutrient availability in aquatic environments (PC3 of BdlC), and (**n**) aeolian dust input fluctuations in BdlC (Zr/Al). Acronyms: LIA: Little Ice Age; MCA; Medieval Climatic Anomaly; DA: Dark Ages; IRHP: Ibero-Roman Humid Period.

Leaf wax biomarkers (*n*-alkanes) and organic proxies from bulk sediments, such as carbon to nitrogen atomic ratio (C/N), hydrogen to carbon atomic ratio (H/C), total organic carbon (TOC), total nitrogen (TN), as well as carbon and nitrogen isotopes (δ^13^C and δ^15^N), are useful tools to understand the origin of the organic matter and the biogeochemical cycles of past environments^36^. Leaf wax extractions from both sedimentary records released enough *n*-alkane concentrations to develop high-quality paleoenvironmental reconstructions (Supplementary Fig. S3). Bulk sediment organic variables were also analysed in BdlC. Previously published bulk sediment organic data from BdlV^20^ will be also used in the environmental models in order to compare similar variables in both studied sites (Supplementary Figs S4, S5). Principal Components Analyses (PCA) were performed using the same variables in both cores: ACL, P_aq_, CPI, C/N, TOC, TN, δ^13^C, and δ^15^N, to identify the different factors (components) that have driven the environmental responses at each site (Fig. 2; Supplementary Figs S6–S8; Supplementary Tables S1–S3). A major shift in environmental parameters, deduced from pollen, algae, and geochemical records, occurred in BdlV at around 5500–5000 cal yr BP, during the transition from an early-middle Holocene lake to a bog^20, 27^ (Supplementary Fig. S5). So, another PCA was performed for the BdlV-bog stage (last 5000 yrs) to compare the bog stages in both areas (Supplementary Fig. S8; Supplementary Table S3). We will focus on the latter in the discussion, because it is more comparable to BdlC.

The PCA analysis yielded three significant components (Supplementary Figs S6–S8; Supplementary Tables S1–S3). The first principal component (PC1) describes 42% of the total variance for BdlC and 56.5% for BdlV-bog. The main positive loadings for PC1 are ACL-CPI-TOC in BdlV-bog, and ACL-CPI-C/N in BdlC, while the main negative component is P_aq_ in both sites. C/N also has a large positive loading in PC1 for both sites. As previously described, P_aq_, ACL, CPI, and C/N are related to the source of organic matter (aquatic/terrestrial), so PC1 reflects the kind of inputs: terrestrial vs aquatic, which we interpret as representing the water availability of the site.

The second principal component (PC2) explains 25% of the total variance for BdlC and 19% for BdlV-bog. The main loadings (positive) for the PC2 are TOC and TN. These variables primarily represent the concentration of organic matter in the sediment^36^, which mostly depends on the terrestrial and aquatic primary productivity and their accumulation rates in the studied sites.

The third principal component (PC3) only describes 14.4% of the total variance in BdlC, and 10.6% in BdlV-bog stage. PC3 in BdlC is mainly related to the isotopes in the bulk sediments and inversely related to wetland plant/algal proxies (low C/N, high TN)^36, 37^, and slightly related to the P_aq_. These relationships suggest that PC3 is likely related to the isotopic enrichment that usually occurs when there is either high aquatic activity/productivity in the water pools and/or C and N limitation^36^. The main loadings in PC3 BdlV-bog are C/N (positive) and TN (negative), related to higher presence of terrestrial vascular plant relative to algal inputs, which mainly depends on the water and nutrient availability.

### Inorganic geochemistry

**Figure 3:**
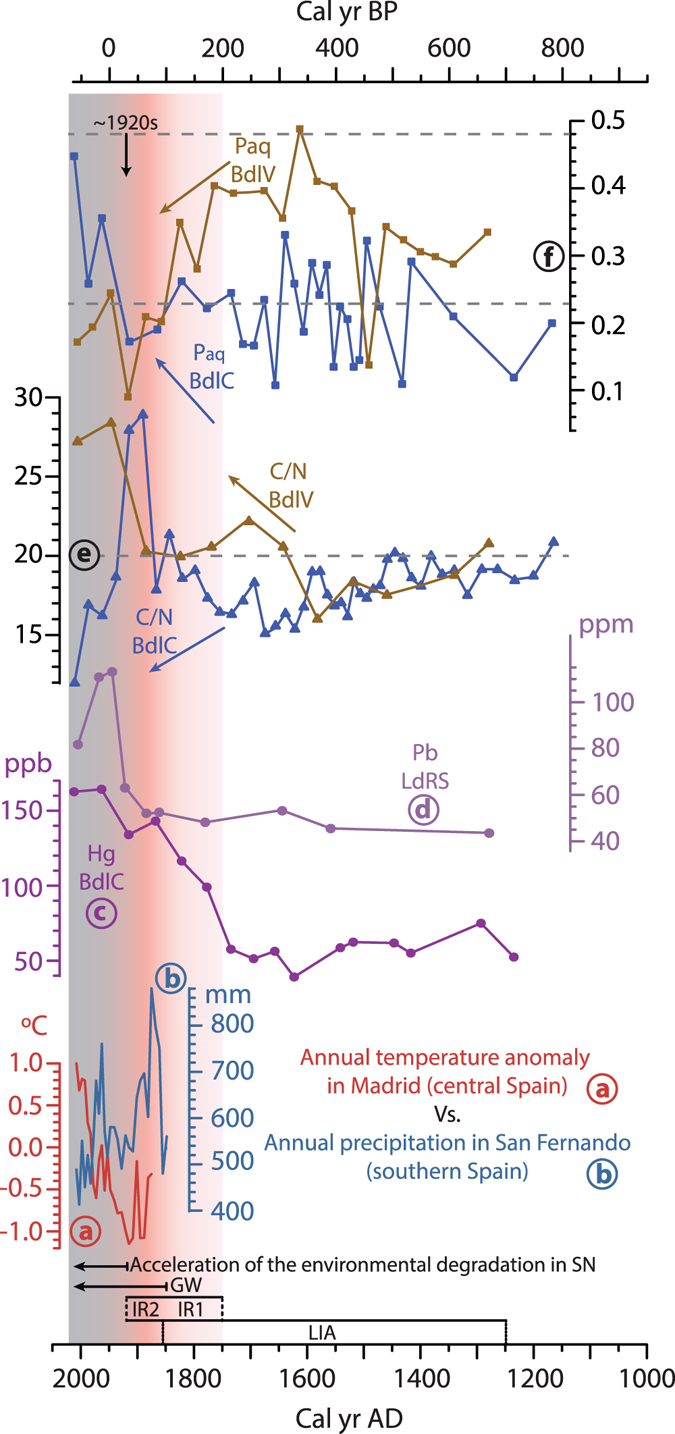
Environmental evolution in Sierra Nevada during the last ∼800 years. (**a**) Longest temperature anomaly record in Spain (Madrid, central Spain)^60^, (**b**) longest precipitation record in Spain (San Fernando, Cádiz, southern Spain)^60^, (**c**,**d**) indirect human impact in the studied area during the last hundred years: (**c**) record of the atmospheric Hg pollution in BdlC (this work), and (**d**) atmospheric Pb pollution in LdRS^13^, (**e**) C/N and (**f**) P_aq_ and fluctuations at BdlC and BdlV. Acronyms: LIA: Little Ice Age; IR1: First Industrial Revolution; IR2: Second Industrial Revolution: GW: Recent Global Warming. P_aq_ thresholds: terrestrial plants <0.23> emergent aquatic plant >0.48 submerged/floating plants^34^. C/N thresholds: algae <10> terrestrial and aquatic vascular plants+algae <20> terrestrial vascular plants^36, 37^.

Concentrations of various metals and elements were measured in the core. For instance, mercury (Hg) is often input to lakes via eolian processes and is typically sourced from industrial and urban emissions^38, 39^. Hg content of the sedimentary record of BdlC is constant (∼55.9 ± 8.9 ppb) before ∼170 cal yr BP, but there is an abrupt increase (to ∼99.1 ppb) since that time, and a generally increasing trend until present, reaching more than 160 ppb (Fig. 3c; Supplementary Fig. S4). In addition, this alpine area is located in the free troposphere, making this location highly sensitive to Saharan uplifted aerosols injected in the troposphere^40, 41^. Certain elemental ratios such as Zr/Th or Zr/Al have been successfully used as a proxy of the source of the aeolian input in Sierra Nevada^10^ and western Mediterranean^42^, as Zr is characteristic of North African rocks, soils, and aerosols^43^. This African Zr signal has also been recorded in summer aerosols at ∼3000 masl in Sierra Nevada, where Zr concentrations of 27.7 ± 3.8 ppm were registered in 2008 AD (Supplementary Table S4). BdlC core shows an increasing trend in the Zr/Al record from ~3500 cal yr BP until the end of the Medieval Climate Anomaly (MCA), ~700 cal yr BP. Since then, there is a fluctuating decreasing trend until the 20^th^ century (Fig. 2n; Supplementary Fig. S4).

### Cyclostratigraphy

Time-series analysis is used to identify important cyclical changes within sediment records. Spectral analyses performed on the multiple proxies at our sites indicate several cycles exist at both cores above the 90%, 95% and 99% confidence threshold (Supplementary Figs S9, S10; Supplementary Table S5). The most significant cycles at BdlC are ∼1500 years for productivity proxies (TOC, TN and TOH) and ∼250–200 years for water availability/nutrient proxies (δ^13^C and δ^15^N, CPI, ACL and P_aq_). The spectral analysis on the BdlV-bog data shows the most significant cycles are at ∼650 and ∼520 years for several productivity and aquatic/terrestrial proxies, and ∼400, ~350–300, and ∼250-year cycles mainly in aquatic/terrestrial inputs (CPI and ACL). The 200–250, and 400-year cycles are typically related to solar-activity^44^, and the others (e.g., 170 or 300-year cycles) have been linked with the North Atlantic Oscillation (NAO)^45^ (Supplementary Table S5). We are aware that the periodicity of the highest frequency cycles (i.e. 113–150 years) might be too short for the resolution of the core data in certain intervals. Although these highest frequency cycles at least double the mean sample spacing for each proxy (Supplementary Figs S9–10) and are over the confidence threshold of 90%, we prefer not to include them in the discussion.

## Discussion

### Bog origin and evolution

Electrical resistivity tomography (ERT) profiles support different origins for each bog (Supplementary Fig. S11). BdlV initially was a lake in a glacial depression during the early Holocene, evolving into a wetland/peatland in the middle-to-late Holocene^20^, ~5100–5000 cal yr BP (Supplementary Fig. S5). BdlC probably had no lacustrine phase, because it exists on a slope. We suggest that this bog stage at both sites resulted from transition to more arid conditions^8, 9, 12^ generated by the demise of the African Humid Period in the western Mediterranean^7^. For BdlV, higher evaporation rates may have precipitated the transition from a small lake to a bog, while increased side slope erosion rates at BdlC valley caused sediment deposition and initial bog substrate formation at this time. At both sites, developing bogs with important biomass production were dominated mainly by vascular terrestrial plants until ∼3600 cal yr BP. Evidence for this exists in the high PC1 values: high C/N (>20), ACL, CPI, and low P_aq_, as well as for the high TN and specially TOC content, the main components of PC2 related to primary production (Fig. 2; Supplementary Figs S4, S5). Thus, predominantly terrestrial conditions are interpreted, especially at BdlC, probably boosted by the high summer insolation^46^. The biomass development in the extreme environments of alpine Sierra Nevada is at present enhanced by warm temperatures or shorter cold seasons, which provide more ice-free surfaces during longer times and greater soil development^21^. High PC2 and TOC values, especially at BdlC, were also recorded throughout the MCA, the last pre-industrial warm era in Europe^47^ (Fig. 2h–k).

After this primary peatland stage (after ~3600 cal yr BP), the general long-trends of the PC1s of BdlC and BdlV-bog during the late Holocene up until ~600–500 cal yr BP tend to be opposite, suggesting different local responses to the same regional climatic oscillations (Fig. 2a–g). The geochemical record of BdlV-bog suggests a shift toward increased aridity and/or a terrestrial environment in this bog through time (Fig. 2d,e; Supplementary Fig. S5), in agreement with the local wetland signal of Cyperaceae pollen at this site, especially during the last millennium^27^. Similar aridity trends deduced from regional pollen data, such as *Pinus* and *Artemisia* are depicted in Sierra Nevada^9, 27, 28^ (Fig. 2a) following the general climatic evolution of the western Mediterranean region during the late Holocene^8, 9, 48^. NAO cycles have been identified by means of spectral analyses in the main loadings of PC1 in BdlV (Supplementary Table S5). This fact along with the moderate-to-high correlation between the NAO index and PC1 in BdlV in different simulations (ranging from r = 0.45 to r = 0.69 and p < 0.01 in 6 out of the 8 developed simulations: Supplementary Table 6), suggest that the water availability variations in BdlV-bog record might have been influenced by NAO fluctuations^45, 49^ (Fig. 2c–e). However, the geochemical record of BdlC points toward greater water availability and/or higher development of aquatic environments at this site during the late Holocene (gradual P_aq_ increase and C/N-ACL-CPI decrease). The local signal of Cyperaceae pollen in BdlC^28^ also confirms this gradual development of local aquatic environments (wetland areas) (Fig. 2b,f,g). The difference in response during this period between both peatlands suggests that, at least for the BdlC record, local conditions may have overridden the regional pattern. For BdlC, local response toward more aquatic conditions during the late Holocene could be related to the increase in the wetland area as a consequence of the progressive infilling of the basin^28^, and it might be also boosted by the Caldera Lake output (as groundwater seepage) (Fig. 1c,d), acting as “water availability buffer” in this site, and preventing or masking the NAO influence and the effect of the general climate evolution in the western Mediterranean. This different response between both adjacent sites also points out the potential problems on single-site studies.

During the last 600 years both PC1s and PC2s (TN and TOC) show fluctuating conditions (Fig. 2d,k), especially during the LIA. While these oscillations are mostly abrupt during this last part of the records, we cannot conclude whether this variability is only caused by the resolution of the records. Although the LIA affected both bogs, BdlV registered the main oscillations, as expected. More humid environments occurred during abrupt and extreme negative NAO excursions at around ~340 and 180 cal yr BP^49^ (Fig. 2c–e). This potential NAO effect along with the drastic fluctuations in the solar irradiance during the LIA^50^ might have given rise to the alternation of periods of longer ice-free conditions vs. periods of longer snow/ice surface cover (changes in the seasonality and precipitation), affecting biomass production proxies, mainly TOC and TN in PC2s (Fig. 2h–k) during this period.

### Allochthonous inputs in the study area

The NW Sahara region has been a persistent source of aeolian input during the Holocene^42^, but the development of commercial agriculture in the Sahel area (∼200 years ago) has largely influenced the aerosol composition in the western Mediterranean^23^. The aeolian inputs to our study area can be estimated by measuring the atmospheric Zr deposition^10, 42^. Saharan aerosols collected at ~3000 masl in the study area of Sierra Nevada confirms this hypothesis (Supplementary Table S4). The Zr/Al ratio in BdlC suggests a continuous increase in the N African dust inputs into the area after ∼4000 cal yr BP, reaching its maximum value around the end of the MCA. After the MCA, these aeolian inputs have been decreasing (with some oscillations) until present (Fig. 2n). According to our simulations, the whole trend of this proxy shows moderate correlations with the NAO index (Supplementary Table S6). However, this correlation declines during the last ∼300 years, pointing to a change in aeolian input features likely caused by either recent major variations in Saharan dust composition^23, 51^, a higher local dust contribution^52^, or some combination.

Allochthonous inputs of phosphorus, calcium, or nitrogen, which are partially ruled by seasonal Saharan dust dynamics^17, 18^, are particularly important at Laguna de la Caldera (upstream of BdlC), which is strongly limited by phosphorus^18^. In that way, phosphorus and calcium cycles in Sierra Nevada reached maximum values during the dry season^17, 18^, and especially during positive NAO index periods^53^, but there is no important correlation between Saharan dust episodes and nitrogen deposition in this region^52^, as it is mainly controlled by rainfall^18, 52^. Similar scenarios are expected in the past; however, this nutrient effect on the water pools cannot be observed during the middle-to-late Holocene transition since terrestrial vascular plants are the main source of organic matter in both sedimentary records (Supplementary Figs S4, S5). After this stage (after ∼3500 cal yr BP), the main loadings of PC3 (δ^13^C and δ^15^N) displayed negative correlations against the aeolian proxy Zr/Al in BdlC (Supplementary Table 6). In these oligotrophic alpine aquatic environments, δ^13^C and δ^15^N values in primary production are mainly influenced by the dissolved inorganic nitrogen (DIN) and dissolved inorganic carbon (DIC) in the water pools, which mainly depends on allochtonous inputs^18, 54^. The inverse correlation between Zr/Al and δ^13^C might be interpreted as Saharan aeolian influence, as Sahara aerosol are carbonate-rich^55^ and might influence the water DIC. On the other hand, the inverse correlation between Zr/Al and δ^15^N is weaker because the nitrogen source in atmospheric depositions is more heterogeneous (dry or wet atmospheric deposition)^18^ (Supplementary Table 6).

### Pre-industrial environmental variability: natural cycles and trends

Environmental changes deduced from marine core pollen data during the middle-to-late Holocene oscillated with a periodicity of ∼1750 yr in the western Mediterranean^56^. Although the BdlC record is too short, this oscillation has not been noted in Sierra Nevada from the longer LdRS^10^ or BdlV records either. This may be because biomass/productivity is affected not only by precipitation variability, as in low altitude sites^56^, but also by temperature seasonality (i.e. length of snow-free season) and nutrient input. Solar cycles and their harmonics are mainly evident in all BdlC geochemical proxies in this study and the previously published pollen record^28^, showing that solar forcing probably influenced locally the environments, at least at centennial scales (Supplementary Table S5). Our data suggest that summer solar irradiance controls biomass development and water availability by modulation of the ice melting/ice-free surfaces and warm summer temperatures. Runoff proxies, related to the dynamic of ice and/or snow melt processes, are also affected by solar cycles (i.e ∼1500-yr cycle) in the nearby record of LdRS^10^. In addition, we identified statistically significant high frequency cycles in the proxies from BdlC that could be related to solar cycles such as the Suess cycle (∼208 years) (Supplementary Table S5).

The general trend of the geochemical record of BdlC points towards greater expansion of wetland environment (more water availability), in agreement with the general increase in the Cyperaceae pollen record at the site^28^ (Fig. 2b,f,g). The water availability here is likely influenced by the buffering effect of Laguna de la Caldera higher up in the catchment basin, which contributes to a subdued response to the NAO relative to the BdlV site. NAO-like cycles are mainly found in the carbon and nitrogen isotopic composition, which are primarily related to the nutrient input/consumption in the BdlC bog (Supplementary Table S5). BdlV record, however, is bound to be influenced by medium frequency NAO-derived cycles (i.e. 650, 520, 300-year cycles) due to the general trend toward drier conditions, agreeing with the regional patterns^9, 10, 20, 27, 28, 57^ (Fig. 2a,d,e). In addition, there is an important influence of the solar cycles in BdlV record (i.e. 400 or 250-year cycle), especially in productivity proxies (i.e. TOC or δ^15^N). Therefore, according to these results, NAO-like cycles seem to control the humidity fluctuation in the area (largely a winter effect), which is also in agreement with the NAO-PC1 correlation in BdlV (Supplementary Table 6), and solar cycles could have regulated the seasonality by modulation of the ice/snow-free surface and temperature during the vegetation-growing season (largely a summer effect).

### Human impact and disruption of natural biogeochemical cycles

Although our study sites are located at high elevation in the protected Sierra Nevada National Park, where direct anthropogenic impact has been minimal, the indirect human impact affecting temperature and precipitation regimes in the region has been significant during the last century^21, 58^. Furthermore, industrial and mining activities at lower elevations have left their footprint in this alpine area, especially as heavy metals (Pb and Hg) in sedimentary records from LdRS^13^ and BdlC, respectively (Fig. 3c,d). These Sierra Nevada records show an increase in the Hg concentration at the end of 18^th^ century (BdlC), following by a rise in the Pb concentration at the beginning of the 20^th^ century (∼1910–1920 AD)^13^ (Fig. 3c,d). Naturally-occurring concentrations of these metals are extremely low in this alpine region, and their primary source has been atmospheric pollution, at least for Pb^13^. Although the main source of the atmospheric Hg pollution is industrial and urban emissions^38, 39^, emissions from local cinnabar (HgS) mining and melting activities at lower elevations in Sierra Nevada appear to have contributed greatly to the local mercury atmospheric pollution, especially during the 18^th^ and 19^th^ centuries. Local mining started between the 17^th^ and 18^th^ centuries, reaching a peak from ∼1885 to ∼1930 AD, ending in 1957^59^ (Fig. 3c). Contrary to the LdRS record, in which the Pb content decreased at around 1970 AD with the use of unleaded fuels^13^, Hg content at BdlC has only stabilised and does not show any significant decrease despite major legal regulations on the industrial use and mining of mercury^38^ (Fig. 3c,d). These persistently high Hg values are related to the increase in industrial activities and fossil fuels, especially coal burning^38^. If this situation continues in the near future, it might be a risk for this protected region, as biological activities can transform mercury into toxic methylmercury in anaerobic wetlands^38^, which would be the case for saturated peat bogs, such as BdlC.

The timing of the industrial development in the area, evidenced by heavy metal records, is coincident with the beginning of abrupt fluctuations in the water resources in both peatland records during the last hundred years. PC1 in BdlC shows a drastic increase in P_aq_ and a decrease in C/N, which points towards more humid environments (Fig. 3e,f). This trend is opposite to the general climatic trends in the western Mediterranean^58^ towards drier conditions^22^ (Fig. 2a). The general trend of PC1 and PC3 from the BdlV-bog, and more specifically the P_aq_ decreases and C/N increases, show a abrupt reduction in the humid environment during the last hundred years until present (Figs 2l and 3e,f). These changes also agree with a lesser NAO influence in the water availability (PC1) of BdlV (Supplementary Table S6) in the last ca. two centuries.

In addition to this amplification of the natural trends during the last ~100–150 years, an important abrupt dry event and development of more terrestrial conditions occurred in the region at ∼1920 AD (Fig. 3b,e,f). This arid event was dramatically recorded in both areas, and is coeval with prevalent NAO positive conditions^49^. The biomass proxies (PC2s) also show significant changes around this time (Fig. 2h–k): (1) the biomass decrease trend is enhanced in BdlC after ∼1920 AD, agreeing with an abrupt decrease in TOC and C/N, and an increase in the P_aq_ and H/C (the latter is also related to algal production), and (2) it had an abrupt and occasional decrease in BdlV, followed by a drastic increase (more terrestrial environments) (Figs 2,h–k and 3e,f; Supplementary Figs S4, S5). All those proxies point towards an important change in the effective precipitation, seasonality, and temperature at the beginning of the 20^th^ century, in agreement with instrumental meteorological data in Spain^60^ (Fig. 3a,b,e,f), reducing the amount of ice/snow on the surface. Coincidentally, this event occurs at the timing of the industrial development of the region at the end of the Second Industrial Revolution (Technological Revolution) in Europe^61^, pointed out by the beginning of the last sharp increase in heavy metal atmospheric pollution caused by human activities (Fig. 3). Similar evolution of the mercury atmospheric pollution during the first part of the 20^th^ century has been identified in the French Alps^39^. This mixture of both natural and human pressure might have been the trigger of the Veleta Glacier melting at the beginning of the 20^th^ century, the last glacier in southern Iberia. This was the first signal of human-induced environmental degradation in Sierra Nevada in the 20^th^ century, pointing at the fact that the natural tolerance threshold in this climate-sensitive area was exceeded.

The trend of consumption of C and N in aquatic environments (PC3) during the last ca. two centuries was also atypical in the BdlC record. Apparently, the important development of algae (C/N and H/C increases) and aquatic environments (P_aq_ increase) at the end of this record did not have any explicit response on PC3, and no enrichment in nitrogen or carbon isotopes was detected (Fig. 2f,g,m; Supplementary Fig. S4). This might have been caused by a change in the source/composition of the nutrient inputs into the area, i.e. increase in local aerosol sources triggered by recent changes in the land use, which could have contributed to dust atmospheric content even during the periods of low Saharan dust inputs^52^. The correlation between the aeolian Zr/Al ratio in BdlC and the NAO index during the last ∼300 years seems to be lower (Supplementary Table S6), suggesting changes in the large-scale atmospheric-controlled dynamics that delivered nutrients in this area. These recent variations in the aeolian source^23^, along with the major anthropogenic disruption on the nitrogen cycle during the last century^62^, might have influenced the composition of the nutrient deposition in our sites, comparing with the late Holocene natural trends, as it has been observed in other mountain wetland areas, such as it is the case of the Alps^24^.

## Conclusions

Unexpected and almost opposite local environmental responses between two nearby alpine bogs during the late Holocene show that the ecosystem development and its response to climate changes is a complex mechanism, even in the same region, and that these responses are highly influenced by the landscape and the environmental evolution of the area. In addition, these opposite environmental responses were amplified during the last centuries. This abrupt amplification at both sites is coincident with regional industrial development, evidenced by the increased heavy metal atmospheric deposition in the study area, as well as the melting and eventual disappearance of the Veleta Glacier at the beginning of the 20^th^ century. Spectral analyses performed on the multiple proxies indicate several cycles exist at both locations that can be explained by a different resilience and sensitivity to climate variations between the peatlands. Anthropogenic influences also appear to moderate the influence of solar and atmospheric cycles in the environments. The obtained paleoenvironmental records indicate that present day ecosystems were settled in the last century after perennial ice disappeared. This means that this former glaciated area will suffer drastic changes and higher vulnerability to climate variations and human-induced environmental pressure if the current climatic trends continue, and can be used as a mirror for endangered still-glaciated areas in Europe.

## Methods

### Site and sampling description

A 56 cm long core was extracted form Borreguil de la Caldera (BdlC 13-01) in 2013 with a Livingstone piston corer. Eighty-two samples for organic analyses in bulk sediment, fifty samples for biomarkers, and eleven samples for inorganic (mercury) analyses were obtained. The age model is based on five calibrated AMS ages^28^ (Supplementary Fig. S1). A 165 cm long core was extracted form Borreguil de la Virgen (BdlV 06-01) in 2006. Ninety-three samples for biomarker analyses were collected. Organic data from bulk sediment of the same record (TOC, C/N, δ^13^C and δ^15^N) were previously published^20^. The age model is based on nine calibrated AMS radiocarbon dates^27^ (Supplementary Fig. S1).

Fifty plant, peat, and soil samples were taken at different distance from the main water pool(s) in several wetlands of Sierra Nevada for biomarker analyses: LdRS (Laguna de Rio Seco, south face, 3020 masl), BdlC (Borreguil de la Caldera, south face 2992 masl), BdlV (Borreguil de la Virgen, north face, 2945 masl), and LdlM (Laguna de la Mula, north face, 2497 masl).

Three high elevation aerosol samples were collected in 2008 by means of 16 MTX1 ARS 1010 automatic deposition sampler in Sierra Nevada (S Spain) at the Sierra Nevada Observatory station (osn: 2896 masl) and the Sierra Nevada Veleta station (vsn: 3000 masl).

### Organic Geochemistry

To track the source of the organic matter in the sediments several proxies have been studied in bulk sediment samples: total organic carbon (TOC), total nitrogen content (TN), atomic C/N ratio, atomic H/C ratio, and carbon and nitrogen isotopes. Three indices of leaf wax biomarkers (*n-*alkanes), assessing the length of the carbon chain length, are used to constrain the source of organic matter and the water availability in the environments: 1) the average chain length (ACL), which is the measurement of the weighted average of the carbon chain lengths; 2) the carbon preference index CPI, which shows the relative abundance of odd vs. even carbon chains, where values lower than 2 point towards even *n-*alkane preference (diagenetic alteration or algae/bacteria influence), and higher than 2, towards odd preference (terrestrial plant source, and thermal immaturity of the source rock)^32^; and 3) the portion aquatic (P_aq_), the ratio between typical aquatic *n-*alkanes and terrestrial ones, which is an useful index to identify aquatic or terrestrial plant sources (water availability) in sedimentary records^34^.

Freeze dried samples from BdlC were decalcified with 1:1 HCl in order to eliminate the carbonate fraction for the organic analyses of bulk sediments. Carbon, nitrogen, and hydrogen content of the decalcified samples were analysed in an elemental analyser Thermo Scientific Flash 2000 at the Centro de Instrumentación Científica (University of Granada). Carbon and nitrogen isotopes (δ^13^C and δ^15^N) were measured simultaneously by means of a continuous flow Isoprime IRMS with a coupled Eurovector EA at the Centro de Instrumentación Científica (University of Granada). Certified Elemental Microanalysis standards were used: Sorgo Flour Standard (δ^13^C: −13.68‰ and δ^15^N: 1.58‰), Wheat Flour Standard (δ^13^C: −27.1‰ and δ^15^N: 2.85‰), and Casein Standard (δ^13^C: −26.98‰ and δ^15^N: 5.94‰), calibrated to the international standards IAEA-CH-6 and IAEA-N1. Isotopic results are expressed in δ notation, using the standard PDB (carbon) and AIR (nitrogen). The atomic C/N ratio has been used in this paper. The calculated precision was better than ±0.1‰ for δ^13^C and δ^15^N.

The total lipid extract from BdlC and BdlV freeze-dried samples was obtained with a 3:1 DCM:methanol solution. After the separation of the neutral and acid fractions by means of aminopropyl-silica gel chromatography using 1:1 DCM:isopropanol and ether with 4% acetic acid respectively, the *n-*alkanes were recovered in the first neutral fraction eluted with hexane trough a 230–400 mesh/35–70 micron silica-gel chromatographic column. The *n-*alkanes were analysed using a GC-FID (Shimadzu 2010) and a GC-MS (Shimadzu OP2010-Plus Mass Spectrometer interfaced with a Shimadzu 2010 GC). To check the reproducibility of the measurements and to quantify the *n-*alkane content, a mixture of *n-*alkanes (C_16_, C_18_, C_19_, C_20_, C_23_, C_25_, C_26_; C_28_; C_30_, C_32_, C_37_) was measured every five samples. The standard reproducibility was better than 97%.

### Inorganic Geochemistry

The potential detrital and aeolian input in the bogs is studied by means of the Zr content in the samples. It has commonly been used as a proxy for aeolian input in different regions, including Saharan dust inputs in the western Mediterranean^10, 42, 43^. Three high elevation aerosol samples were also selected to check their Zr content. Firstly, they were weighed and digested with HNO_3_-HF mixture at 120 °C overnight. After drying down, each sample was treated with HNO_3_ several times. Samples were re-dissolved in a diluted HNO_3_, and trace element compositions were measured by external calibration method with an ICP_MS NEXION 300D quadrupole inductive coupled plasma-mass spectrometry (ICP-MS) at the Centro de Instrumentación Científica (University of Granada, Spain). Procedural blank was nearly negligible for all elements presented in this study.

An Avaatech X-Ray fluorescence (XRF) core Scanner was used to obtain high-resolution Zr/Al profiles in the BdlC core at the XRF-Core Scanner Laboratory (University of Barcelona, Spain). Two runs of analyses were performed: one at 10 s count times, 10 kV X-ray voltage, and 650 mA X-ray current for lighter elements (Al), and another one at 35 s count time, 30 kV X-ray voltage, and 1700 mA X-ray current for heavier elements (Zr). Among all the obtained signals, we have focused on the Zr/Al ratio, as it is the most relevant one for the purpose of this paper. Results were expressed in intensities (counts per second, cps) and normalized for the total sum in cps in every measure.

Total mercury concentrations in 11 samples from the uppermost 26 cm of BdlC were determined to track the potential heavy metal pollution at high elevation. They were analysed by means of an Advanced Mercury Analyser (LECO AMA-254) with an absolute mass detection limit of 0.01 ng of Hg^63^. Samples were combusted in an oxygen-rich atmosphere (99.5%) and the evolved gasses were transported via an oxygen carrier gas through specific catalytic compounds to a gold-plated ceramic, which collects the mercury in vapour. The amalgamator was heated up to approx. 700 °C to release mercury to the detection system. The working range was between 0.05 ng and 500 ng. In this study, samples of peat bog and quality control materials with masses of 20 mg to 100 mg were inserted into the AMA-254 spectrometer in a nickel boat, dried at 120 °C for 50 s, combusted in the oxygen atmosphere at 700 °C for 150 s and after 45 s of waiting (the time needed for cleaning of the system) the next sample was introduced. The entire analytical procedure was validated by analysing certified reference material DORM-3 (Fish tissue, NRCC, Canada) at the beginning and end of each set of samples, ensuring that the instrument remained calibrated during the analytical routine.

### Statistics

Statistical treatment of the data was performed by means of PAST free software^64^. Data were normalised subtracting the mean and dividing by the standard deviation to conduct the Principal Component Analyses (PCA). Spectral analyses have also been carried out with PAST and the REDFIT module. The time series has been fitted to an AR(1) red noise model, and 90%, 95%, and 99% confidence levels were chosen. The selected cycles range from around 1/3 of the total time interval (~1500 yr), in the case of the lowest frequency cycles, to at least two times the estimated time represented for the most usual sampling interval (mean sample spacing), which depends on the site and the studied variables (Supplementary Figs S9 and S10)^10^.

## Electronic supplementary material

Supplemenetary Information
