# Supplementary material for: Alpine bogs of southern Spain show human-induced environmental change superimposed on long-term natural variations
Source: Sci Rep. 2017 Aug 7;7:7439. doi: 10.1038/s41598-017-07854-w (PMC5547100; doi:10.1038/s41598-017-07854-w)
Supplement: Supplementary file 1 — Supplemenetary Information [file 41598_2017_7854_MOESM1_ESM.pdf]

Supplementary Information

Alpine bogs of southern Spain show human-induced environmental change superimposed on long-term natural variations

Antonio García-Alix, Francisco J. Jiménez-Espejo, Jaime L. Toney, Gonzalo Jiménez-Moreno, María J. Ramos-Román, R. Scott Anderson, Patricia Ruano, Ignasi Queralt, Antonio Delgado Huertas, and Junichiro Kuroda

The supplementary material file includes:

Supplementary Figures S1 – S11

Supplementary Tables S1 – S6

Supplementary Methods

Supplementary References

Supplementary Figures

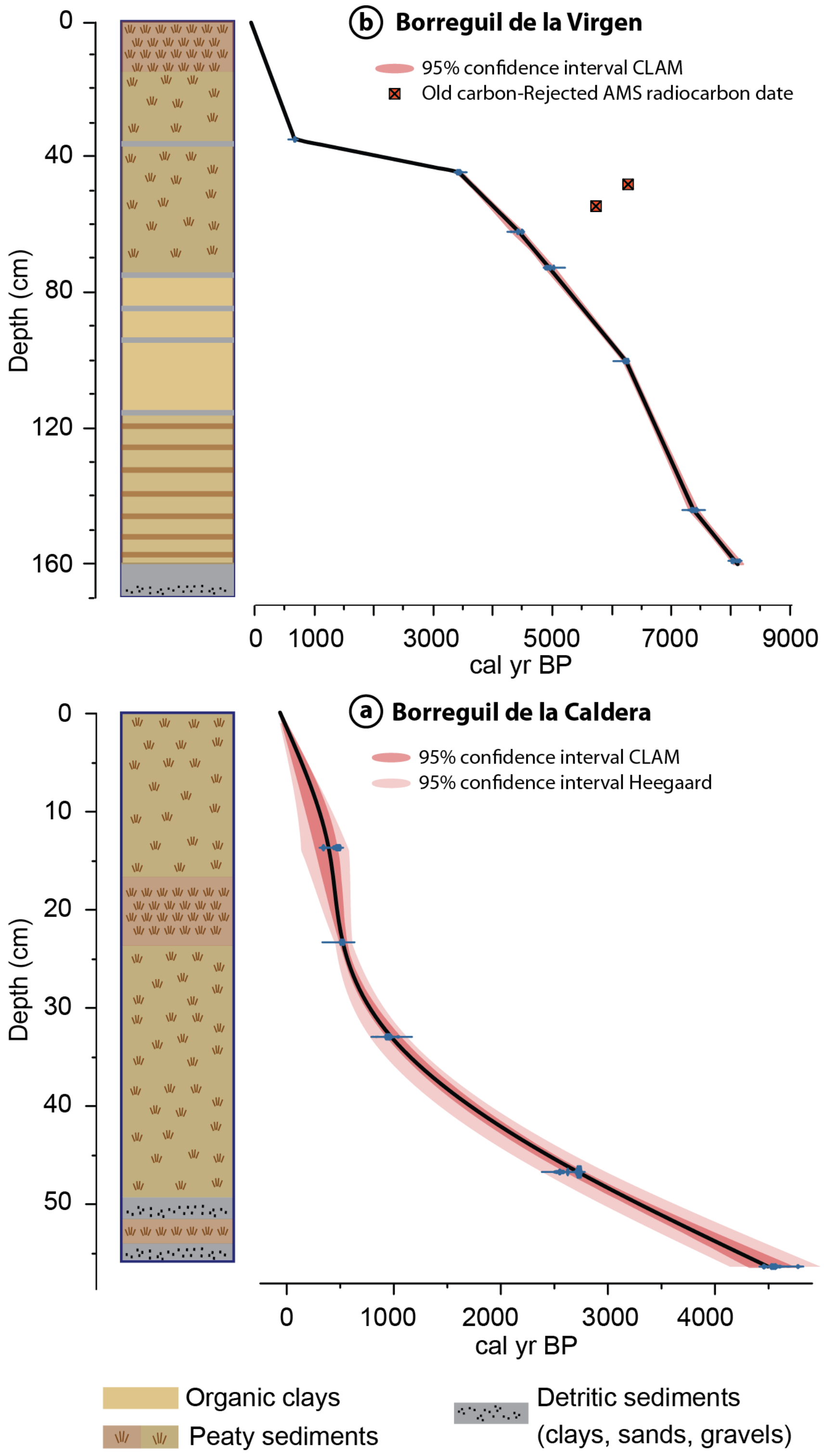

Supplementary Figure S1. **Age models and sedimentary columns of the studied sites.** (**a**) BdlC age model from Ramos Román et al. (2016)[1] using both Heegaard model[2] and Clam software(http://www.chrono.qub.ac.uk/blaauw/clam.html)[3]. (**b**) BdlV age model from Jiménez Moreno and Anderson (2012)[4] using Clam software[3].

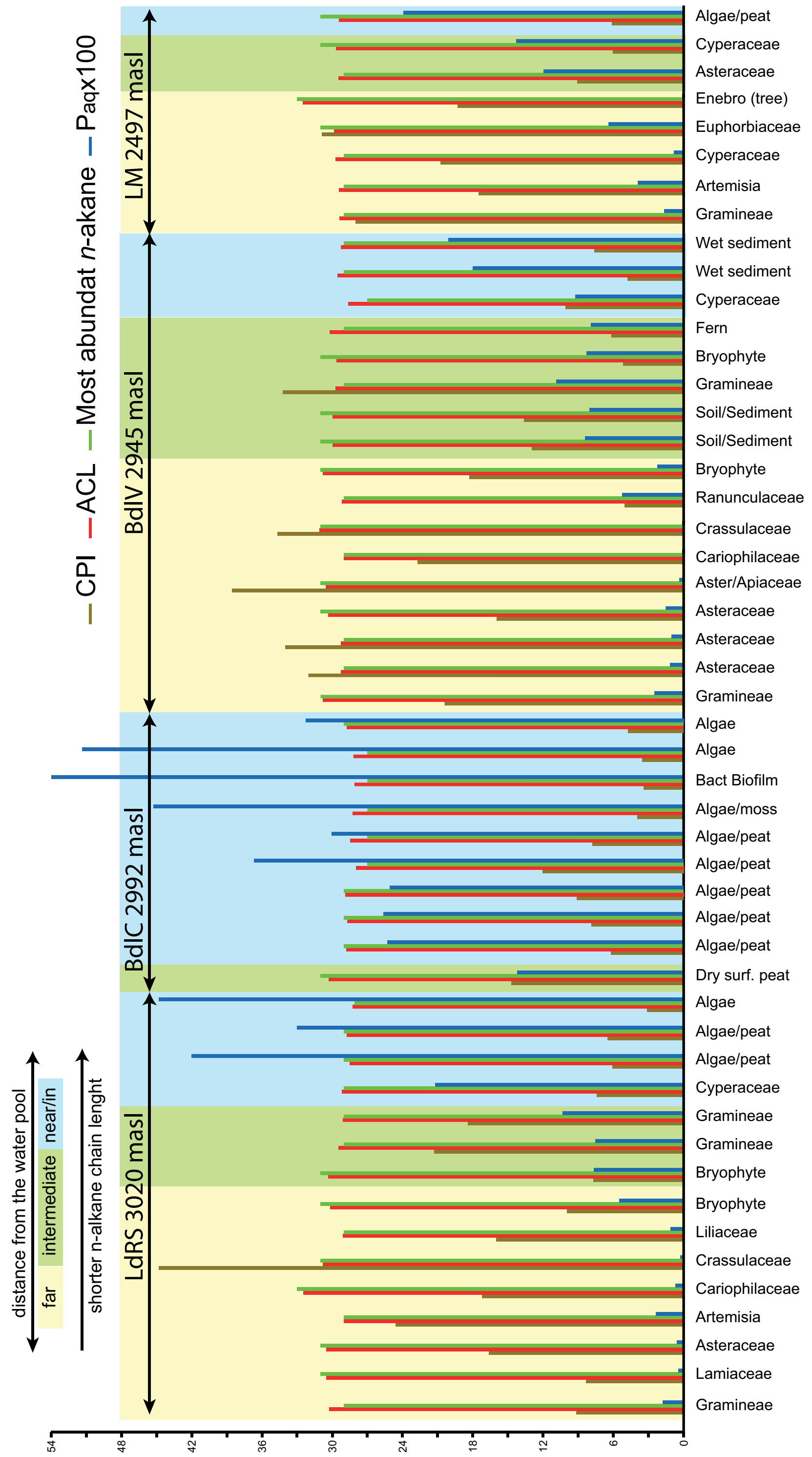

Supplementary Figure S2. **Biomarker results in recent plants and soils.** *n*-alkane indices (CPI, ACL, P_{aq}, and the most important *n*-alkane in each sample) from the studied plant, algae, and peat samples at different distance from the water pools at four different sites in Sierra Nevada.

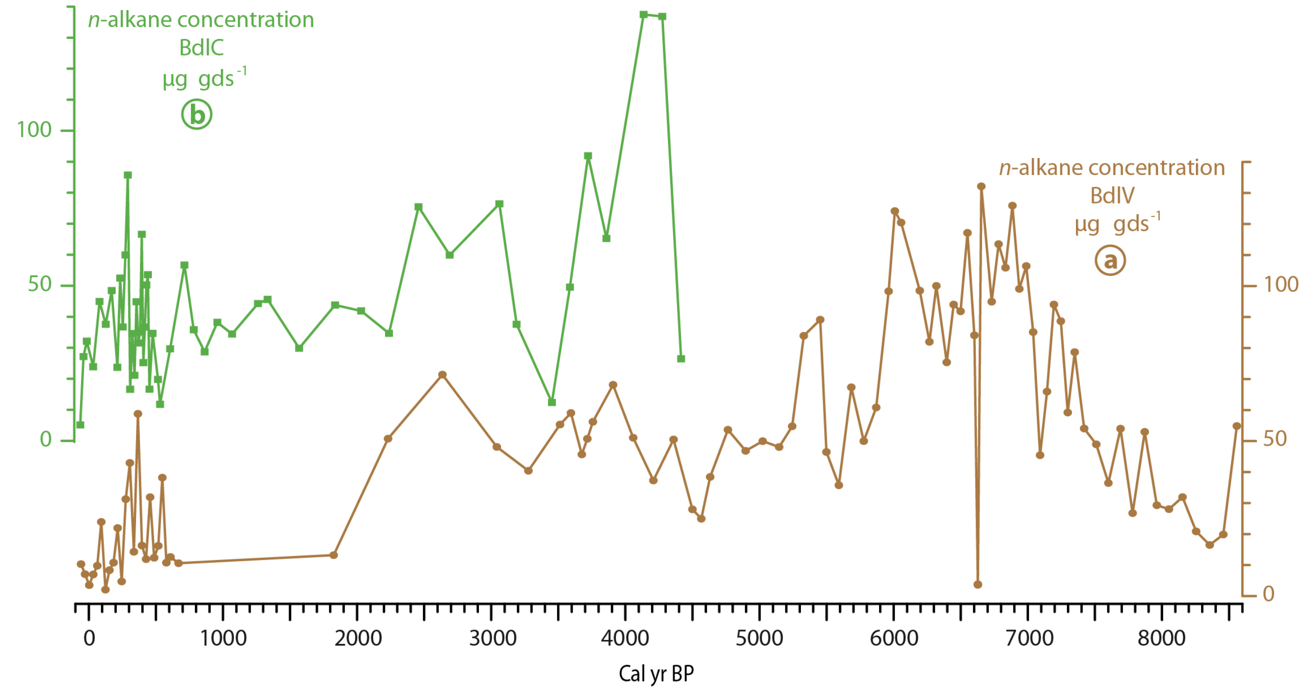

Supplementary Figure S3. ***n*-alkane concentration in the studied records.** *n*-alkane concentrations in microgram of sediment per gram of dry sample (µg gds^{-1}) from (**a**) BdlV and (**b**) BdlC. The highest *n*-alkane concentration in BdlV occurred from ~7000 to ~6000 cal yr BP, with values usually higher than 100 µg gds^{-1} (gds = gram of dry sediment). Subsequently, values generally fluctuated between 25 and 89 µg gds^{-1} until ~2200 cal yr BP. Similar fluctuations occurred in BdlC from ~4500 to 2200 cal yr BP, but with higher *n*-alkane concentrations, ranging from 12 to 137 µg gds^{-1}. The concentrations are more constant in BdlC until the Little Ice Age (LIA). During the Little Ice Age (LIA) higher fluctuations occurred in both records, which subsequently declined (BdlC: 5-45 µg gds^{-1} and BdlV: 2-24 µg gds^{-1}).

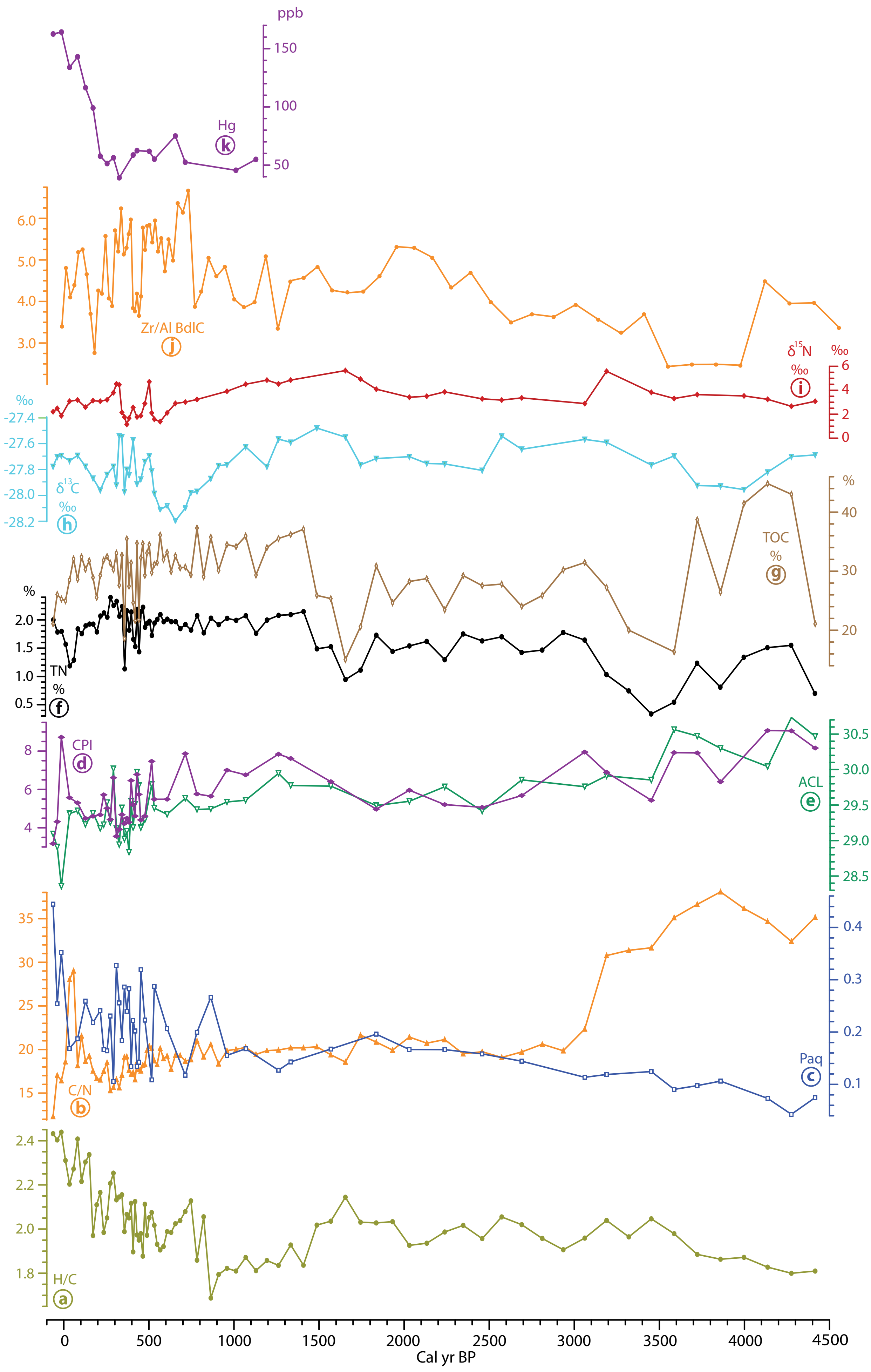

ppb
150
100
50
Hg
k
6.0
5.0
4.0
3.0
Zr/Al BdIC
j
δ15N
‰
‰
6
4
2
0
i
‰
-27.4
-27.6
-27.8
-28.0
-28.2
δ13C
‰
h
%
40
30
20
TOC
%
g
%
2.0
1.5
1.0
0.5
TN
%
f
CPI
d
8
6
4
ACL
e
30.5
30.0
29.5
29.0
28.5
35
30
25
20
15
C/N
b
0.4
0.3
0.2
0.1
Paq
c
2.4
2.2
2.0
1.8
H/C
a
0
500
1000
1500
2000
2500
3000
3500
4000
4500
Cal yr BP

Supplementary Figure S4. Organic proxies studied from the BdlC record during the middle-to-late Holocene: (**a**) atomic hydrogen – carbon ratio (H/C), (**b**) atomic carbon – nitrogen ratio (C/N), (**c**) portion aquatic (P_{aq}), (**d**) carbon preference index (CPI), (**e**) average chain length (ACL), (**f**) total nitrogen content (TN%), (**g**) total organic carbon (TOC%), (**h**) carbon isotopic composition of the bulk organic matter ($\delta^{13}C$), (**i**) nitrogen isotopic composition of the bulk organic matter ($\delta^{15}N$), (**j**) zirconium – aluminium ratio, and (**k**) mercury concentration (ppb).

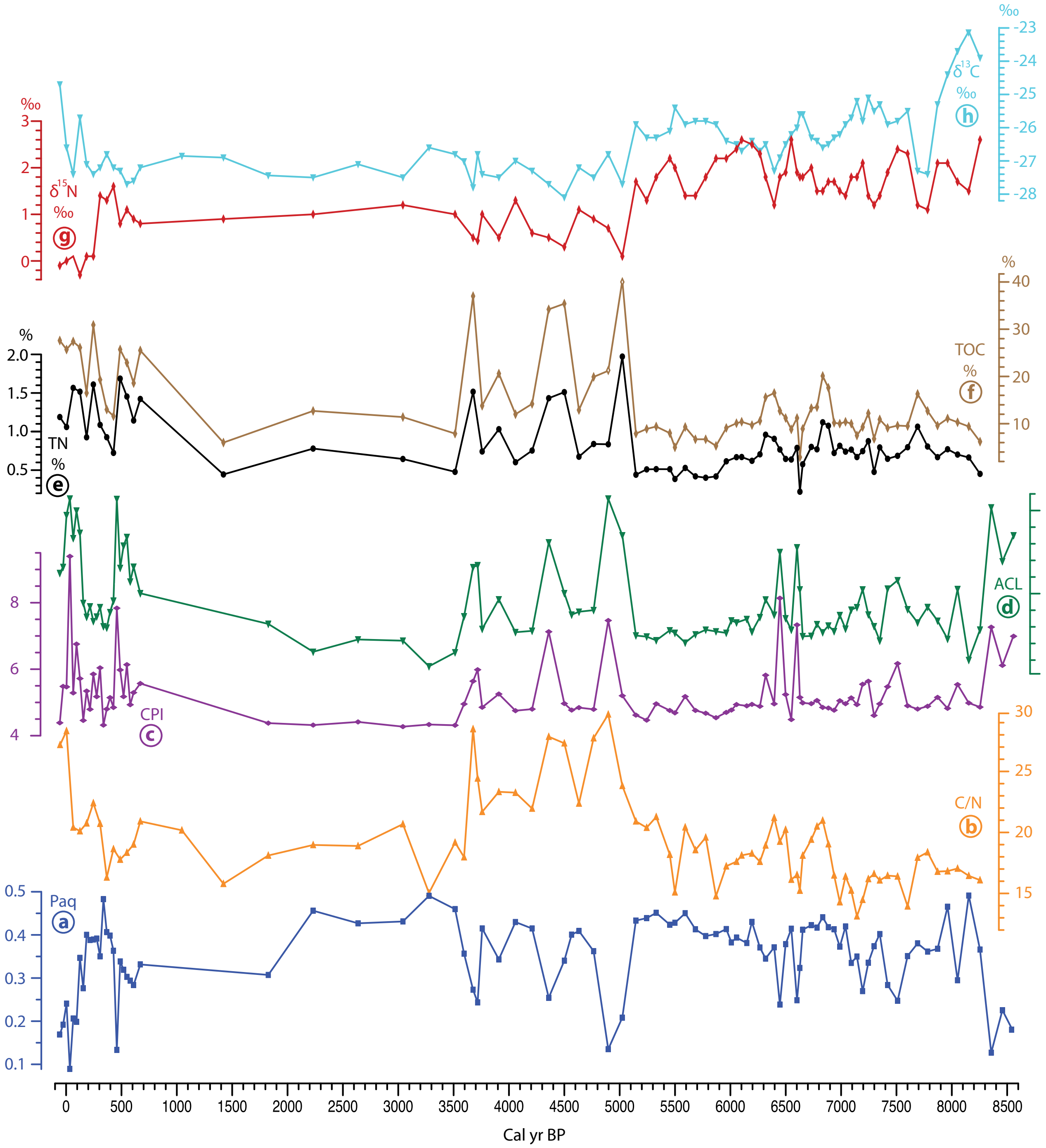

Supplementary Figure S5. Organic proxies studied from the BdlV record during the Holocene: (**a**) portion aquatic (P_{aq}), (**b**) atomic carbon – nitrogen ratio (C/N)[5], (**c**) carbon preference index (CPI), (**d**) average chain length (ACL), (**e**) total nitrogen content (TN%), (**f**) total organic carbon (TOC%)[5], (**g**) nitrogen isotopic composition of the bulk organic matter ($\delta^{15}N$)[5], and (**h**) carbon isotopic composition of the bulk organic matter ($\delta^{13}C$)[5].

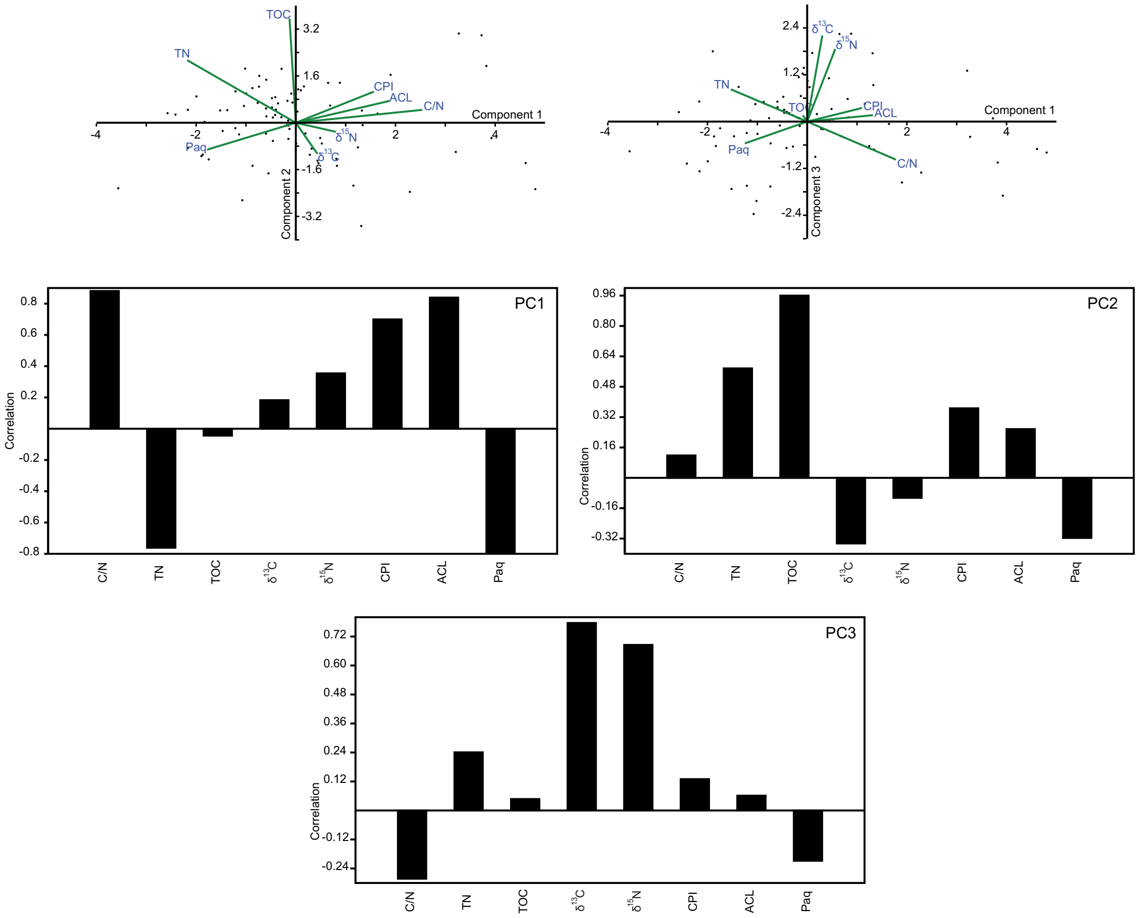

Supplementary Figure S6. Organic PCA results from Borreguil de la Caldera. Used indices ACL, P_{aq}, CPI, C/N, TOC, TN, $\delta^{13}C$, and $\delta^{15}N$. Biplot figure and loadings (correlation) of the most important PCs: PC1, PC2, and PC3 in BdlC record.

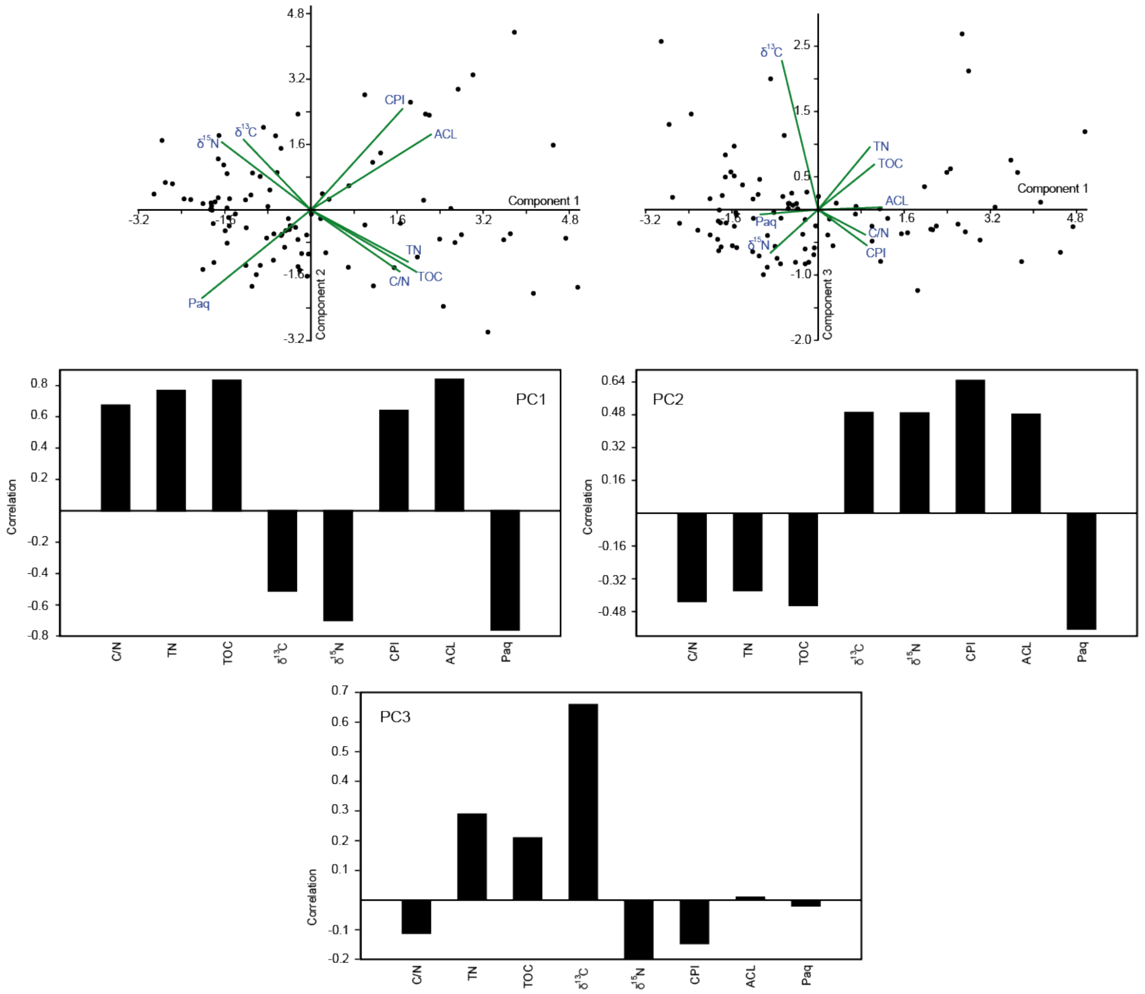

Supplementary Figure S7. Organic PCA Results from Borreguil de la Virgen (whole record). Used indices ACL, P_{aq}, CPI, C/N, TOC, TN, $\delta^{13}C$, and $\delta^{15}N$. Biplot figure and loadings (correlation) of the most important PCs: PC1, PC2, and PC3 in BdlV record.

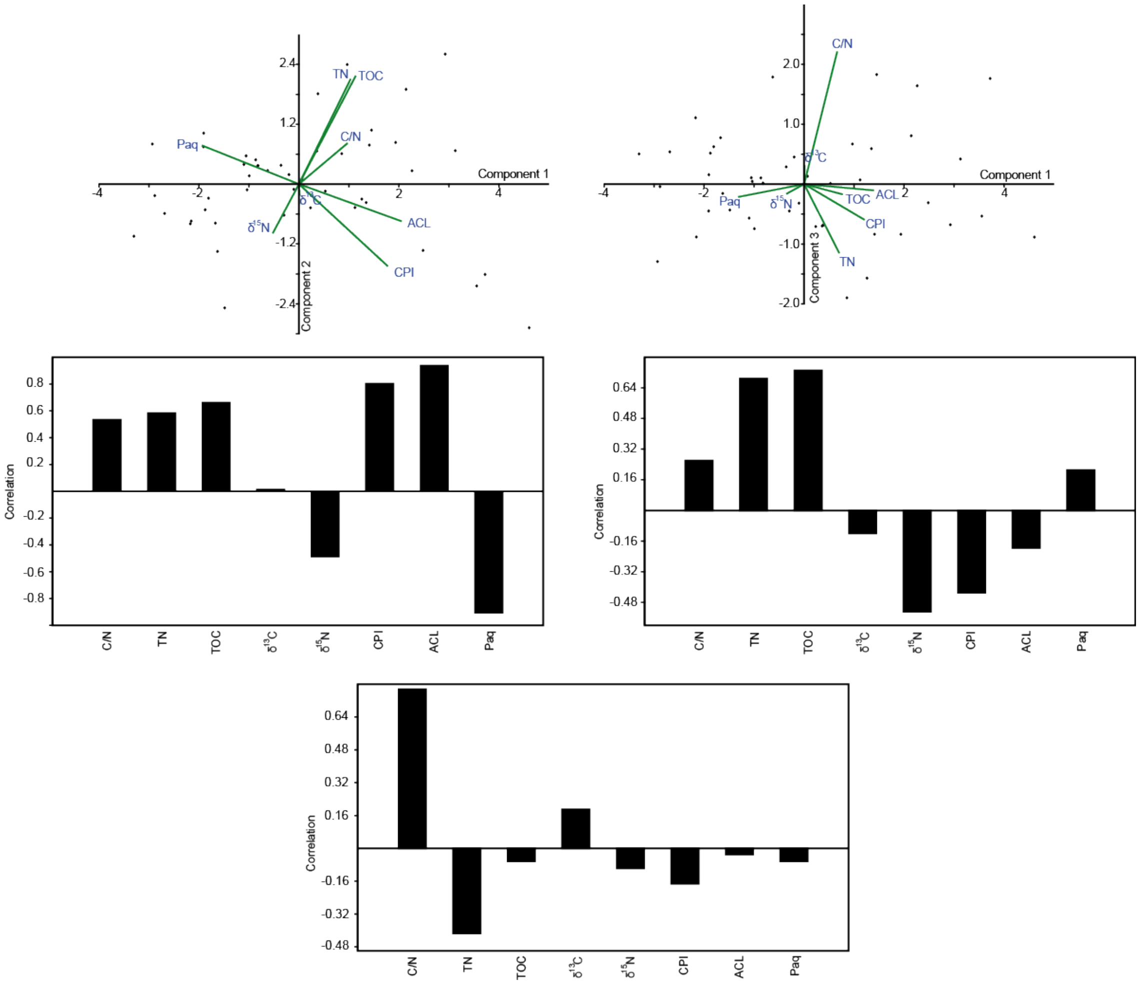

Supplementary Figure S8. Organic PCA Results. Borreguil de la Virgen-bog stage (last 5000 years). Used indices ACL, P_{aq}, CPI, C/N, TOC, TN, $\delta^{13}C$, and $\delta^{15}N$. Biplot figure and loadings (correlation) of the most important PCs: PC1, PC2, and PC3 in BdlV-bog record.

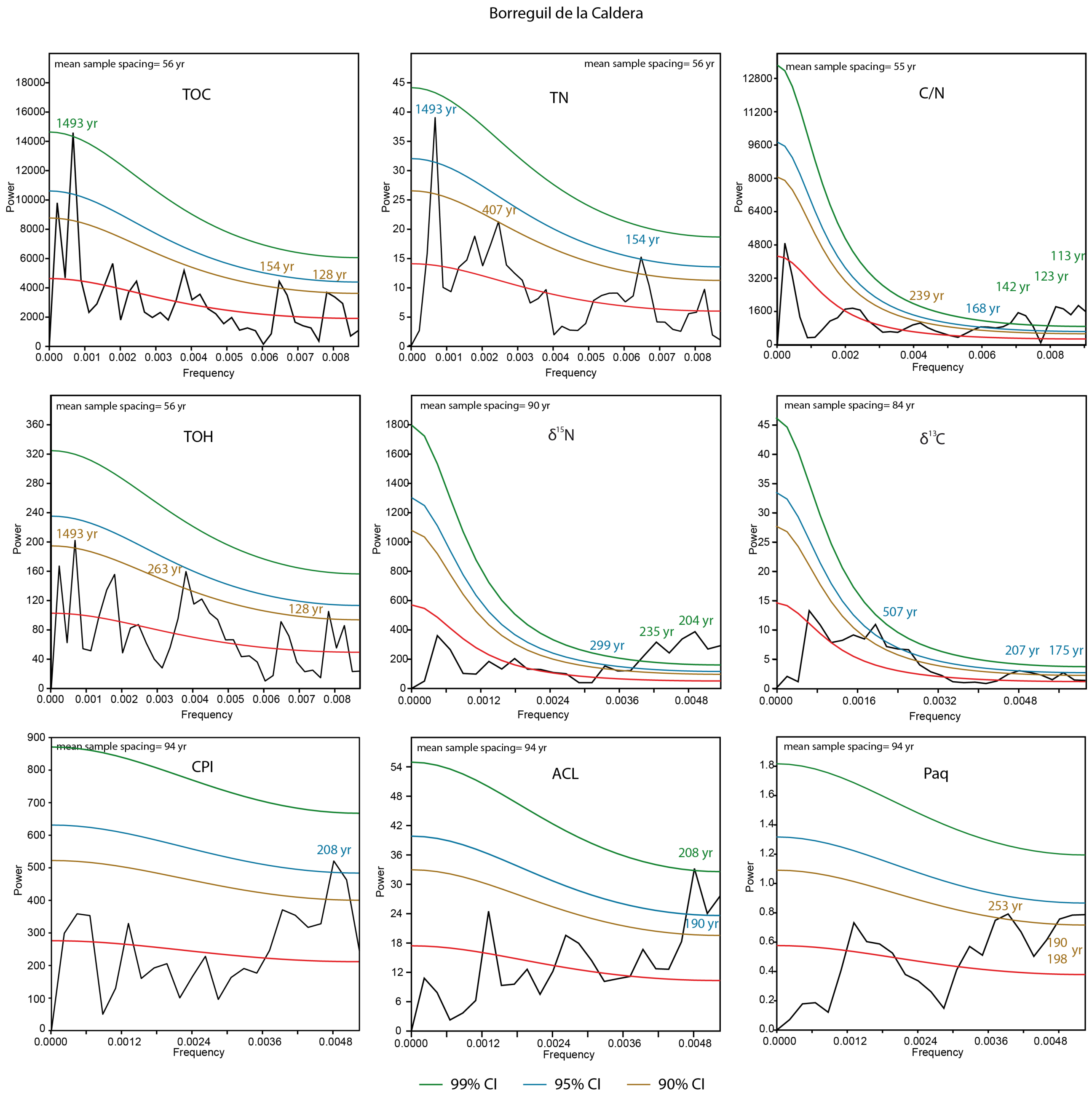

Supplementary Figure S9. Spectral analyses of the organic proxies of BdlC. AR(1) red noise (red line). Confidence thresholds: 90% (brown line), 95% (blue line), and 99% (green line).

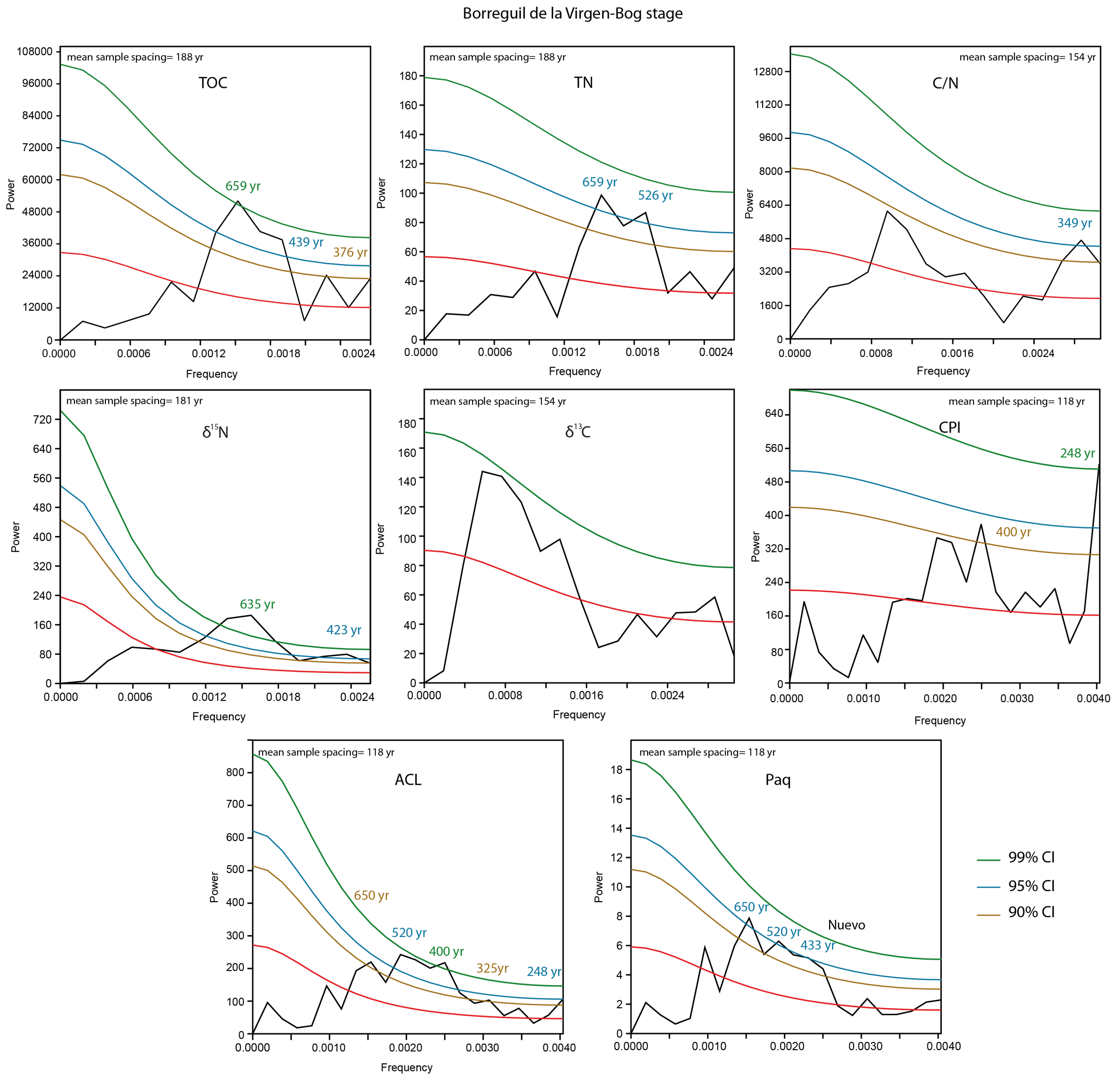

Supplementary Figure S10. Spectral analyses of the organic proxies of BdlV-bog stage. AR(1) red noise (red line). Confidence thresholds: 90% (brown line), 95% (blue line), and 99% (green line).

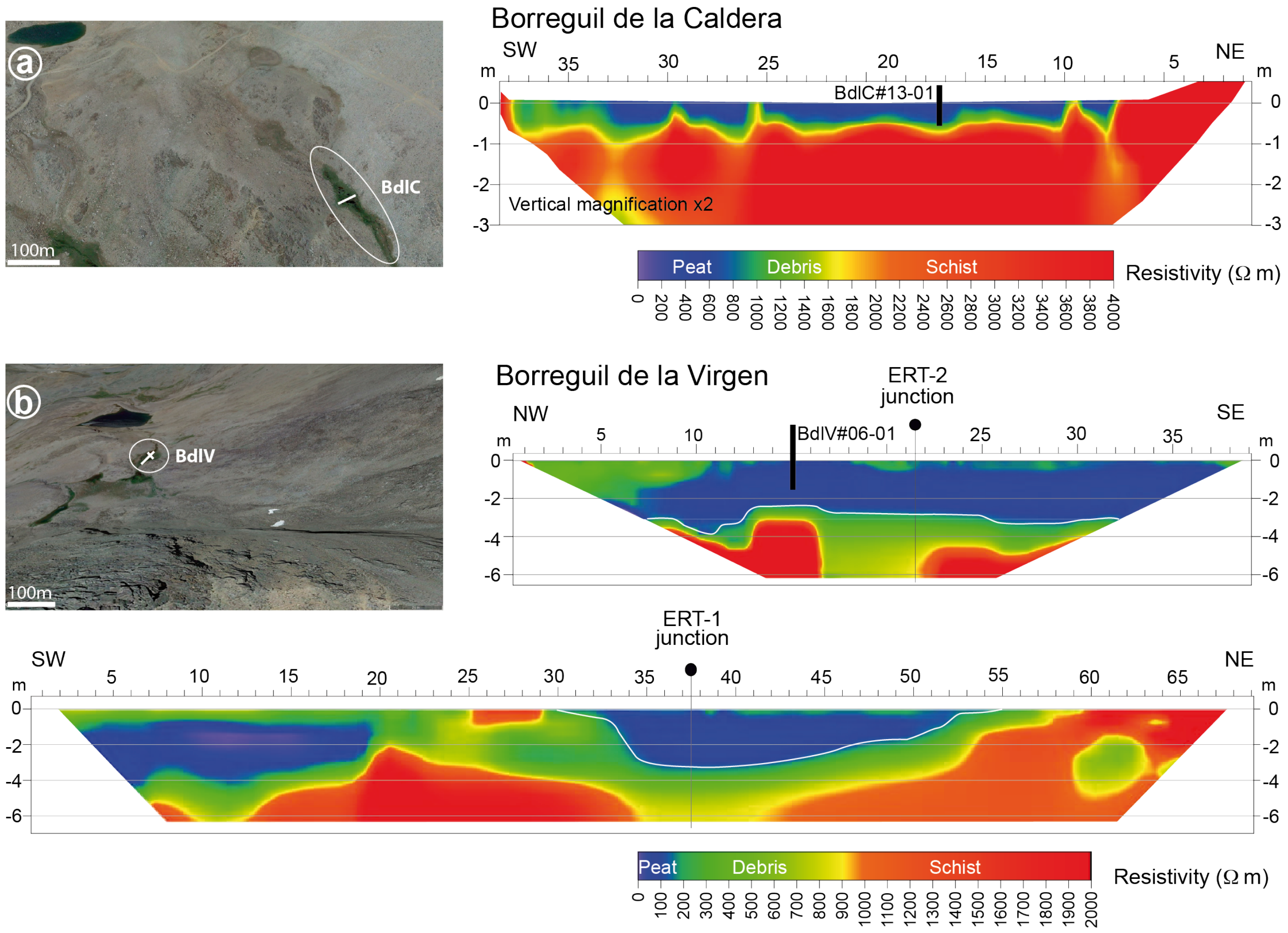

Supplementary Figure S11. Electrical resistivity tomographies (ERT) of BdlC (**a**) and BdlV (**b**) showing the subsurface distribution of electrical resistivity of the basins where the sedimentary records were taken. The profiles ERT-1 and ERT-2 are perpendicular in BdlV. Both horizontal and vertical scales are in meters. Resistivity gradients: peat < 800ohm.m-scouring schist < 1800ohm.m-basement schist in BdlC and peat < 150ohm.m-scouring schist < 1000ohm.m-basement schist in BdlV. Depth estimation of the peat/lake deposits in the studied sites deduced from the ERT: ~0.6m in BdlC and ~2.2-2.3m in BldV. Note that peats from BdlC are completely pure water-saturated (more water availability), causing high resistivity values of up to 600 ohm.m, while the less saturated peat at BldV (less water availability) has standard resistivity values for peat deposits (100 ohm.m[6]). Data source and software: (**a**), and (**b**) maps from Google Earth Pro [7.1.5.1557] (https://www.google.es/earth/download/gep/agree.html) using the data provided by Google 2016 and DigitalGlobe 2016. ERT profiles were designed with Surfer® [11] from Golden Software, LLC (www.goldensoftware.com).

Supplementary Tables

BdlC PCs	Eigenvalue	% Variance	Cumulative variance
**1**	2.6	41.9	
**2**	1.5	25.0	66.9
**3**	0.9	14.4	81.3
**4**	0.4	7.0	88.3
**5**	0.4	6.0	94.4
**6**	0.2	3.5	97.9
**7**	0.1	1.5	99.4
**8**	0.0	0.6	100.0

Supplementary Table S1. Organic PCA Results from Borreguil de la Caldera. Eigenvalue, and percentage of variance explained with the different Principal Components in BdlC record.

BdlV-w PCs	Eigenvalue	% variance	Cumulative variance
**1**	3.6	52.3	52.3
**2**	1.7	25.4	77.7
**3**	0.5	7.5	85.2
**4**	0.5	6.7	91.9
**5**	0.3	3.8	95.7
**6**	0.2	3.2	98.9
**7**	0.1	1.0	4.2
**8**	0.0	0.1	100.0

Supplementary Table S2. Organic PCA Results from Borreguil de la Virgen (whole record). Eigenvalue, and percentage of variance explained with the different Principal Components in BdlV whole record.

BdlV-b PCs	Eigenvalue	% variance	Cumulative variance
**1**	3.8	56.6	
**2**	1.3	19.0	75.5
**3**	0.7	10.6	86.2
**4**	0.4	6.7	92.9
**5**	0.2	3.6	96.5
**6**	0.1	1.9	98.4
**7**	0.1	1.3	3.3
**8**	0.0	0.2	100.0

Supplementary Table S3. Organic PCA Results from Borreguil de la Virgen (last 5000 years). Eigenvalue, and percentage of variance explained with the different Principal Components in BdlV-bog record.

Sample	Location	Elevation (masl)	Zr (ppm)
osn-01 Aerosol	37º03'N 3º23'W	2896	30.2
osn-03 Aerosol	37º03'N 3º23'W	2896	29.5
vsn-01 Aerosol	37°17'N 3°11'W	3000	23.3

Supplementary Table S4. Zr content (ppm) in aerosol samples from high elevation observatories in Sierra Nevada (S Spain).

**Site**	**Proxy**	**Cycles (years)**										
		**~1500**	**~650**	**~520**	**~400**	**~350**	**~300**	**~250**	**~200**	**~170**	**~150**	**130-113**
**BdlC**	**TOC**	99%									90%	90%
	**TN**	95%			90%						95%	
	**C/N**							90%		95%	99%	99%
	**TOH**	90%						90%				90%
	**$\delta^{15}N$**						95%	99%	99%			
	**$\delta^{13}C$**			95%					95%	95%		
	**CPI**								95%			
	**ACL**								99%			
	**Paq**							90%	90%			
**BdlV bog stage**	**TOC**		99%		95%–90%							
	**TN**		95%	95%								
	**C/N**					95%						
	**$\delta^{15}N$**		99%		95%							
	**$\delta^{13}C$**											
	**CPI**				90%			99%				
	**ACL**		90%	95%	99%	90%		95%				
	**Paq**		95%	95%								
**Forcing**		S/ A-O/ Or	S/ NAT/ NAO	S/ NAO	S	S/ NAO	NAO	S	S: Suess	NAO	S: Gleissberg band	

Supplementary Table S5. Summary of the cycles obtained from the spectral analyses from BdlC and BdlV(bog stage) records. Acronyms: S, Solar; A-O; Atmosphere-Ocean circulation; Orbital forcing; NAT: North Atlantic thermohaline circulation; NAO, North Atlantic Oscillation. The *150-113-year cycles*, likely Gleissberg cycles, have been identified as solar cycles. They have a characteristic frequency between 60 and 150 years during the middle and late Holocene[7]. Nevertheless, Gleissberg cycles are only clear at BdlC; however, they are in the detection limit due to the sampling spacing (red and orange colours). BdlC also preserved a *~1500-year cycle,* not present at BdlV. This *~1500-year cycle* is quite common in marine and terrestrial records in the Northern Hemisphere, and its origin is quite controverted: solar, atmospheric-ocean circulation, or orbital modulation[8-10], or even a mixture of the 1000- and 2000-year solar cycles[11]. The Suess (or de Vries) *cycle at 208 year*[12,13], related to solar fluctuations, is one of the most important cycle

identified in Holocene records[13], and can affect lacustrine paleoproductivity and summer temperatures, such as in some records of Alaska[14,15]. The *170* and *300-year cycles* are related to NAO fluctuations[16]. The *250-year cycle* has been identified as a solar cycle during the last 2000 years[17]. The *350-year cycle* has been recently described as solar forced[18,19], that could also influence the NAO cycles[20]. Obtained *~400-year cycle* can be considered a solar cycle, and has impact in the late Holocene humidity from north America to China[21,22]. The *~520-year cycle* can correspond to the ~500-530-year solar activity cycle[23]. Those changes in solar irradiance induce NAO anomalies[20,24]. It has also been related to warm/cold fluctuations during the middle/late Holocene in East Asia[24], and variations in the North Atlantic circulation patterns[25]. *650-year cycle* has been recognised in the storminess frequency, seasonal sea-ice development, and cooling trends at high latitudes in the Northern Hemisphere, in agreement with solar radiation fluctuations[26,27]. Those variations at high latitudes could be linked with NAO variations[28]. It has been also proposed as a secondary harmonic of the *1300-year cycle*[29] related to North Atlantic thermohaline circulation and/or sea surface temperatures[29]. This is the first mention of the 650-year cycle in continental records, so a mechanism connecting terrestrial and marine environments, such as the NAO, should drive this cycle.

**Correlation**	**Simulation**	**Scenario 1 3500 cal yr BP – present**		**Scenario 2 3500 – 250 cal yr BP**		**Scenario 3 4500 cal yr BP – present**		**Scenario 4 4500 – 250 cal yr BP**	
**NAO – PC1 BdlV**	A	0.52	p<0.01	0.69	p<0.01	0.45	p<0.01	0.54	p<0.01
	B	0.58	p<0.01	0.73	p<0.01	0.51	p<0.01	0.60	p<0.01
	C	0.46	p=0.022	0.74	p<0.01	0.37	p=0.042	0.53	p<0.01
	D	0.69	p<0.01	0.79	p<0.01	0.55	p<0.01	0.60	p<0.01
**NAO – Zr/Al BdlC**	A	0.49	p<0.01	0.56	p<0.01	0.45	p<0.01	0.51	p<0.01
	B	0.56	p<0.01	0.58	p<0.01	0.53	p<0.01	0.58	p<0.01
	C	0.39	p=0.057	0.54	p=0.012	0.40	p=0.027	0.52	p<0.01
	D	0.48	p=0.024	0.54	p=0.012	0.52	p<0.01	0.56	p<0.01
**Zr/Al – δ^{13}C BdlC**	A	-0.64	p<0.01	-0.64	p<0.01				
	B	-0.58	p<0.01	-0.67	p<0.01				
	C	-0.65	p<0.01	-0.79	p<0.01				
	D	-0.78	p<0.01	-0.77	p<0.01				
**Zr/Al – δ^{15}N BdlC**	A	-0.35	p=0.036	-0.33	p=0.060				
	B	-0.33	p=0.050	-0.32	p=0.070				
	C	-0.51	p=0.012	-0.49	p=0.026				
	D	-0.46	p=0.031	-0.38	p=0.090				

Supplementary Table S6. Simulations of proxy correlations in four different scenarios. As data spacing was different in all these studied proxies, a linear interpolation was performed in order to obtain time series equally spaced (100 and 150 years). Afterwards, the mobile average was worked out along the time series (taking into account either the 3 nearest points or the 5 nearest points) in order to easily identify trends by means of smoothing out data irregularities. Simulations: A) regular interpolation of 100 years sampling spacing and 3 data points moving average. B) regular interpolation of 100 years sampling spacing and 5 data points moving average. C) regular interpolation of 150 years sampling spacing and 3 data points moving average. D) regular interpolation of 150 years sampling spacing and 5 data points moving average. In addition, four different scenarios have been considered: Scenario 1) from 3500 cal yr BP (after the initial bog development) to the present. Scenario 2) from 3500 cal yr BP to 250 cal yr BP (the same time interval as Scenario 1, but without the most recent period of enhanced human influence). Scenario

3) from 4500 cal yr BP (including the initial bog development) to the present. Scenario 4) from 4500 cal yr BP to 250 cal yr BP (the same time interval as Scenario 2, but without the most recent period of enhanced human influence). Note that during the main bog development (4500-3500 cal yr BP) there was no correlation between Zr/Al – $\delta^{13}C$ BdlC and Zr/Al – $\delta^{15}N$ BdlC.

Supplementary Methods

Electrical resistivity tomography. ERT calculates the subsurface distribution of electrical resistivity[30], allowing subsurface lithologic patterns to be deduced. Three profiles of electrical resistivity tomography (ERT) were conducted to understand the origin and evolution of the wetlands. The three profiles were acquired with a 4 and 12-channel multiple gradient electrode array in two combined protocols: GRAD4LX8 and GRAD4S8 with a Terrameter SAS 4000 and Terrameter LS equipments (ABEM, Inc.). Considering the accuracy and depth penetration target, electrodes were deployed at 0.5 m, reaching a penetration depth of 6 m. The obtained apparent resistivity pseudosections were inverted using Res2dinv software (v. 3.56; Geotomo Inc.) with a standard least-square inversion method[31], a model refinement constraints, and the same parameters and treatment flow for all models.

Supplementary References

1 Ramos-Román, M. J. *et al.* Centennial-scale vegetation and North Atlantic Oscillation changes during the Late Holocene in the southern Iberia. *Quaternary Science Reviews* **143**, 84-95 (2016).

2 Heegaard, E., Birks, H. J. B. & Telford, R. J. Relationships between calibrated ages and depth in stratigraphical sequences: an estimation procedure by mixed-effect regression. *The Holocene* **15**, 612-618, doi:doi:10.1191/0959683605hl836rr (2005).

3 Blaauw, M. Methods and code for 'classical' age-modelling of radiocarbon sequences. *Quaternary Geochronology* **5**, 512-518, doi:http://dx.doi.org/10.1016/j.quageo.2010.01.002 (2010).

4 Jiménez-Moreno, G. & Anderson, R. S. Holocene vegetation and climate change recorded in alpine bog sediments from the Borreguiles de la Virgen, Sierra Nevada, southern Spain. *Quaternary Research* **77**, 44-53, doi:10.1016/j.yqres.2011.09.006 (2012).

5 García-Alix, A., Jiménez-Moreno, G., Anderson, R. S., Jiménez Espejo, F. J. & Delgado Huertas, A. Holocene environmental change in southern Spain deduced from the isotopic record of a high-elevation wetland in Sierra Nevada. *Journal of Paleolimnology* **48**, 471-484, doi:10.1007/s10933-012-9625-2 (2012).

6 Asadi, A. & Huat, B. B. Electrical resistivity of tropical peat. *Electronic Journal of Geotechnical Engineering,* **14**, 1-9 (2009).

7 Ma, L. H. Gleissberg cycle of solar activity over the last 7000 years. *New Astronomy* **14**, 1-3, doi:http://dx.doi.org/10.1016/j.newast.2008.04.001 (2009).

8 Bond, G. *et al.* Correlations between climate records from North Atlantic sediments and Greenland ice. *Nature* **365**, 143-147 (1993).

9 Debret, M. *et al.* The origin of the 1500-year climate cycles in Holocene North-Atlantic records. *Clim. Past* **3**, 569-575, doi:10.5194/cp-3-569-2007 (2007).

10 Jiménez-Espejo, F. J. *et al.* Saharan aeolian input and effective humidity variations over western Europe during the Holocene from a high altitude record. *Chemical Geology* **374-375**, 1-12, doi:10.1016/j.chemgeo.2014.03.001 (2014).

11 Obrochta, S. P., Miyahara, H., Yokoyama, Y. & Crowley, T. J. A re-examination of evidence for the North Atlantic "1500-year cycle" at Site 609. *Quaternary Science Reviews* **55**, 23-33, doi:http://dx.doi.org/10.1016/j.quascirev.2012.08.008 (2012).

12 Sonett, C. P. & Suess, H. E. Correlation of bristlecone pine ring widths with atmospheric 14C variations: a climate-Sun relation. *Nature* **307**, 141-143 (1984).

13 Stuiver, M. & Braziunas, T. F. Sun, ocean, climate and atmospheric 14CO2 : an evaluation of causal and spectral relationships. *The Holocene* **3**, 289-305, doi:10.1177/095968369300300401 (1993).

14 Hu, F. S. *et al.* Cyclic Variation and Solar Forcing of Holocene Climate in the Alaskan Subarctic. *Science* **301**, 1890-1893, doi:10.1126/science.1088568 (2003).

15 Wiles, G. C., D'Arrigo, R. D., Villalba, R., Calkin, P. E. & Barclay, D. J. Century-scale solar variability and Alaskan temperature change over the past millennium. *Geophysical Research Letters* **31**, n/a-n/a, doi:10.1029/2004GL020050 (2004).

16 Olsen, J., Anderson, N. J. & Knudsen, M. F. Variability of the North Atlantic Oscillation over the past 5,200 years. *Nature Geosciences* **5**, 808-812, doi:http://www.nature.com/ngeo/journal/v5/n11/abs/ngeo1589.html - supplementary-information (2012).

17 Vaquero, J. M., Gallego, M. C. & García, J. A. A 250-year cycle in naked-eye observations of sunspots. *Geophysical Research Letters* **29**, 58-51-58-54, doi:10.1029/2002GL014782 (2002).

18 Summerhayes, C. P. *Earth's Climate Evolution.* 416 (Wiley-Blackwell, 2015).

19 Steinhilber, F. *et al.* 9,400 years of cosmic radiation and solar activity from ice cores and tree rings. *Proceedings of the National Academy of Sciences* **109**, 5967-5971, doi:10.1073/pnas.1118965109 (2012).

20 Lamy, F., Arz, H. W., Bond, G. C., Bahr, A. & Pätzold, J. Multicentennial-scale hydrological changes in the Black Sea and northern Red Sea during the Holocene and the Arctic/North Atlantic Oscillation. *Paleoceanography* **21**, n/a-n/a, doi:10.1029/2005PA001184 (2006).

21 Wu, J., Yu, Z., Zeng, H. A. & Wang, N. Possible solar forcing of 400-year wet–dry climate cycles in northwestern China. *Climatic Change* **96**, 473-482, doi:10.1007/s10584-009-9604-4 (2009).

22 Yu, Z. & Ito, E. Possible solar forcing of century-scale drought frequency in the northern Great Plains. *Geology* **27**, 263-266, doi:10.1130/0091-7613(1999)027<0263:psfocs>2.3.co;2 (1999).

23 Stuiver, M., Grootes, P. M. & Braziunas, T. F. The GISP2 δ18O Climate Record of the Past 16,500 Years and the Role of the Sun, Ocean, and Volcanoes. *Quaternary Research* **44**, 341-354, doi:http://dx.doi.org/10.1006/qres.1995.1079 (1995).

24 Xu, D. *et al.* 500-year climate cycles stacking of recent centennial warming documented in an East Asian pollen record. *Scientific Reports* **4**, 3611, doi:10.1038/srep03611 http://www.nature.com/articles/srep03611 - supplementary-information (2014).

25 Chapman, M. R. & Shackleton, N. J. Evidence of 550-year and 1000-year cyclicities in North Atlantic circulation patterns during the Holocene. *The Holocene* **10**, 287-291, doi:10.1191/095968300671253196 (2000).

26 Sarnthein, M. *et al.* Centennial-to-millennial-scale periodicities of Holocene climate and sediment injections off the western Barents shelf, 75°N. *Boreas* **32**, 447-461, doi:10.1111/j.1502-3885.2003.tb01227.x (2003).

27 Berner, K. S., Koç, N., Godtliebsen, F. & Divine, D. Holocene climate variability of the Norwegian Atlantic Current during high and low solar insolation forcing. *Paleoceanography* **26**, n/a-n/a, doi:10.1029/2010PA002002 (2011).

28 Bader, J. *et al.* A review on Northern Hemisphere sea-ice, storminess and the North Atlantic Oscillation: Observations and projected changes. *Atmospheric Research* **101**, 809-834, doi:http://dx.doi.org/10.1016/j.atmosres.2011.04.007 (2011).

29 Rodrigo-Gámiz, M., Martínez-Ruiz, F., Rodríguez-Tovar, F. J., Jiménez-Espejo, F. J. & Pardo-Igúzquiza, E. Millennial- to centennial-scale climate periodicities and forcing mechanisms in the westernmost Mediterranean for the past 20,000 yr. *Quaternary Research* **81**, 78-93, doi:http://dx.doi.org/10.1016/j.yqres.2013.10.009 (2014).

30 Daily, W., Ramirez, A., Binley, A. & LeBrecque, D. Electrical resistance tomography. *The Leading Edge* **23**, 438-442 (2004).

31 Loke, M. H. *Tutorial: 2-D and 3-D electrical imaging surveys.* (Geotomo Software, Inc, 2010).
